# TEAD4 Regulates apical domain homeostasis and cell-positioning to maintain the trophectoderm lineage during mouse blastocyst development

**DOI:** 10.1098/rsob.250441

**Published:** 2026-10-07

**Authors:** Rebecca Collier, Martina Bohuslavová, Michaela Vaškovičová, Aleksandar I. Mihajlović, Andrea Hauserová, Valeriya Zabelina, Monika Fluks, Lenka Gahurová, Dávid Drutovič, Alexander W. Bruce

**Affiliations:** ^1^ Department of Molecular Biology and Genetics (Faculty of Science), University of South Bohemia in Ceske Budejovice, Ceske Budejovice, South Bohemian Region, Czech Republic; ^2^ Institute of Animal Physiology and Genetics CAS, Liběchov, Central Bohemian Region, Czech Republic; ^3^ Centre de Recherche du Centre Hospitalier de l'Universite de Montreal, Montreal, Quebec, Canada

**Keywords:** tead4, trophectoderm, inner cell mass, polarization, apical domain, cell internalization, actin

## Abstract

In mammalian preimplantation embryos, distinct cell lineages occupy defined spatial niches. The outer trophectoderm (TE) forms an epithelial monolayer surrounding the inner cell mass (ICM) and blastocyst cavity. In mice, the *Tead4* gene encodes a polarity-dependent transcription factor required for TE specification. Here, we demonstrate that *Tead4* also preserves TE integrity during blastocyst maturation. Clonal siRNA-mediated *Tead4* knockdown (KD) disrupts outer cell apical domain morphology, causing abnormal allocation of these clones to an enlarged ICM, despite minimal impact on established polarity. Fixed sample and live embryo light-sheet microscopy imaging reveal that TEAD4-deficient outer cells atypically migrate into the blastocyst ICM, sometimes via apical domain abscission, or are repositioned post-division, linking disrupted apical domain morphology to altered spatial positioning and fate. Transcriptomic analysis indicates that TEAD4 regulates genes involved in cytoskeletal organization, particularly those related to actin and cell adhesion, which we propose are critical for maintaining outer cell TE positioning. Combined KD of *Tead4* and two of its targets, the atypical GTPases *Rnd1* and *Rnd3*, partially rescues misallocation but does not prevent apical morphological abnormalities. These findings indicate TEAD4 and its downstream regulatory network actively contribute to blastocyst TE maintenance, beyond initial specification, up until the peri-implantation stage, ensuring proper lineage segregation.

## 1. Introduction

During early mouse embryo development, three distinct lineages form within the blastocyst. The trophectoderm (TE), which later develops into the foetal component of the placenta, is a surface layer of epithelial cells that surrounds a fluid-filled cavity and an inner cell mass (ICM). The primitive endoderm (PrE) arises within the ICM, forming a monolayer at its interface with the cavity, and eventually contributes to the yolk sac endoderm. The epiblast (EPI) consists of pluripotent cells located deep inside the ICM and serves as the source of cells for the future fetus [1–4].

The first cell fate decision begins at the eight- to 16-cell stage and continues through the 16- to 32-cell stage. During this time, cells that are polarized along their apical-basolateral axis become actively segregated from inner unpolarized cells and ultimately reside on the outer surface of the embryo. This segregation results in the formation of the polarized TE on the outside and an apolar ICM on the inside. Thus, the ICM is composed of primary and secondary cells generated during the subsequent rounds of cell cleavage division [5,6], although an occasional contribution of cells internalized in a third round of division has been reported [7]. The second cell fate decision occurs later within the blastocyst ICM, where cells either specify as the EPI or differentiate into the PrE [8–10]. After cell cleavage, the spatial positioning of 16- and 32-cell stage blastomeres is linked to the unequal inheritance of outer cell actomyosin-driven apical constriction. Increased contractility causes some cells to internalize [11,12], in a process closely associated with apical-basolateral polarity [13–17]. Outer cells lacking polarity proteins or with experimentally depleted polarity factors internalize into the ICM [18–20], indicating that inherited polarity acts to inhibit contractility and cell internalization [11,12]. Maintaining outer cell polarity is central to TE specification, as the Hippo pathway effector AMOT is sequestered to contactless apical domains enriched in polarity factors, preventing YAP1 phosphorylation. Unphosphorylated YAP1 translocates to the nucleus and forms TEAD4–YAP1 complexes, transcriptionally activating TE-related genes, such as *Cdx2*. By contrast, apolar inner cells exhibit active Hippo signalling, leading to cytoplasmic retention of phospho-YAP1, failure to activate TE genes [21,22] and maintenance of pluripotency [23]. Additionally, asymmetric inheritance of keratin intermediate filaments has been reported to stabilize the outer cell apical cortex, further promoting Hippo pathway suppression and TE fate [24].

Our previous work showed that RNAi knockdown (KD) of *Tead4* not only impairs TE differentiation, aligning with genetic knockout (KO) models [25,26], but also leads to increased contribution of *Tead4* KD clones to the late (E4.5) blastocyst ICM. Immunofluorescence revealed that these supernumerary ICM cells predominantly expressed EPI transcription factor profiles rather than those of PrE cells, suggesting that TEAD4-mediated TE-specification cues prime later PrE differentiation within the ICM [27]. Notably, these effects occurred without any overt polarity defects. However, apical-basolateral polarity initiation at the eight-cell stage is reported to involve the partially redundant roles of the *Tead4* and *Tfap2c* genes and the small GTPase RHOA [16]. Collectively, while TEAD4 promotes TE differentiation by inducing genes, such as *Cdx2*, through polarity-dependent Hippo signalling suppression [21,22], these data also indicate that TEAD4 may actively maintain TE-fated blastomeres in outer positions, supporting the ‘polarity-dependent cell-positioning model’ [28] and prompting further investigation in this study.

We found that clonal *Tead4* KD in preimplantation mouse embryos leads to an atypical allocation of outer cell clones to the ICM, which peaks during blastocyst maturation after the 32-cell stage. This is not associated with the formation of apolar outer cells, though the expression of the apical polarity protein PARD6B [29,30] is reduced and the expression of other Hippo signalling components is also reduced, indicating a degree of functional TEAD4-mediated feedback. Rather, such cellular misallocations result from a combination of asymmetric cell divisions and apical domain constriction. In addition, we report mechanisms of unconventional cell division associated with the formation of apical domain blebs or the abscission of apical domains that result in cell internalization independent of cell division. RNA-Seq analysis revealed significant transcriptome differences between *Tead4* KD clones and their non-targeted sister outer cells, mainly in genes related to the cytoskeleton and actin organization, including cell adhesion. Targeting the identified actin-related atypical small GTPases *Rnd1* and *Rnd3* candidate genes partially rescued the *Tead4* KD-induced internalization phenotypes, although the induced apical domain distortions persisted. These findings show *Tead4* plays a novel role in maintaining apical domain stability and outer cell spatial positioning, ensuring TE-specified cells remain appropriately positioned during blastocyst maturation.

## 2. Results

### 2.1. Clonal *Tead4* knockdown induces atypical outer-to-inner cell allocation and distorted apical domain morphology from the 32-cell stage

Previously, we found that microinjecting two-cell stage mouse embryos with *Tead4* dsRNA reduces the contribution of confirmed *Tead4* KD clones to the PrE lineage and biases them towards EPI in late blastocysts (E4.5). We interpreted this as evidence that TEAD4 primes PrE fate in secondary ICM founders derived from outer 16-cell stage blastomeres, by forming active TEAD4–YAP1 complexes via polarity-dependent Hippo suppression, which are inhibited in the *Tead4* KD clones. Additionally, *Tead4* KD clones (comprising 50% of the embryo) were more frequently located in the E4.5 ICM than the outer TE, resulting in larger ICMs [27]. These findings suggest that TEAD4 not only drives TE specification/differentiation [21,22], but also maintains proper outer positioning of TE-specified blastomeres; a hypothesis we explore further in this study.

We repeated the two-cell stage microinjection RNAi strategy, co-injecting a *Tead4*-specific siRNA (si*Tead4*) together with histone H2B–RFP mRNA, producing embryos with both fluorescently marked *Tead4* KD and unmarked control clones. We performed the same approach using a non-targeting control siRNA (siNTC) as a control. Embryos were cultured for 48, 54, 60 or 72 hours (equivalent to E3.5, E3.75, E4.0 and E4.5), fixed, immunofluorescently (IF) stained and imaged by confocal microscopy to assess clonal contributions to outer positions and the ICM (figure 1A). At all stages, si*Tead4* injections effectively abolished TEAD4 protein expression in marked clones (figure 1B,C; supplementary material, figure S1A,B). We also performed whole-embryo *Tead4* KD, followed by quantitative reverse-transcription PCR (RT-qPCR) analysis at E3.5 (supplementary material, figure S1D–F; table S2), additionally validating the efficiency of the si*Tead4* construct.

**Figure 1.**
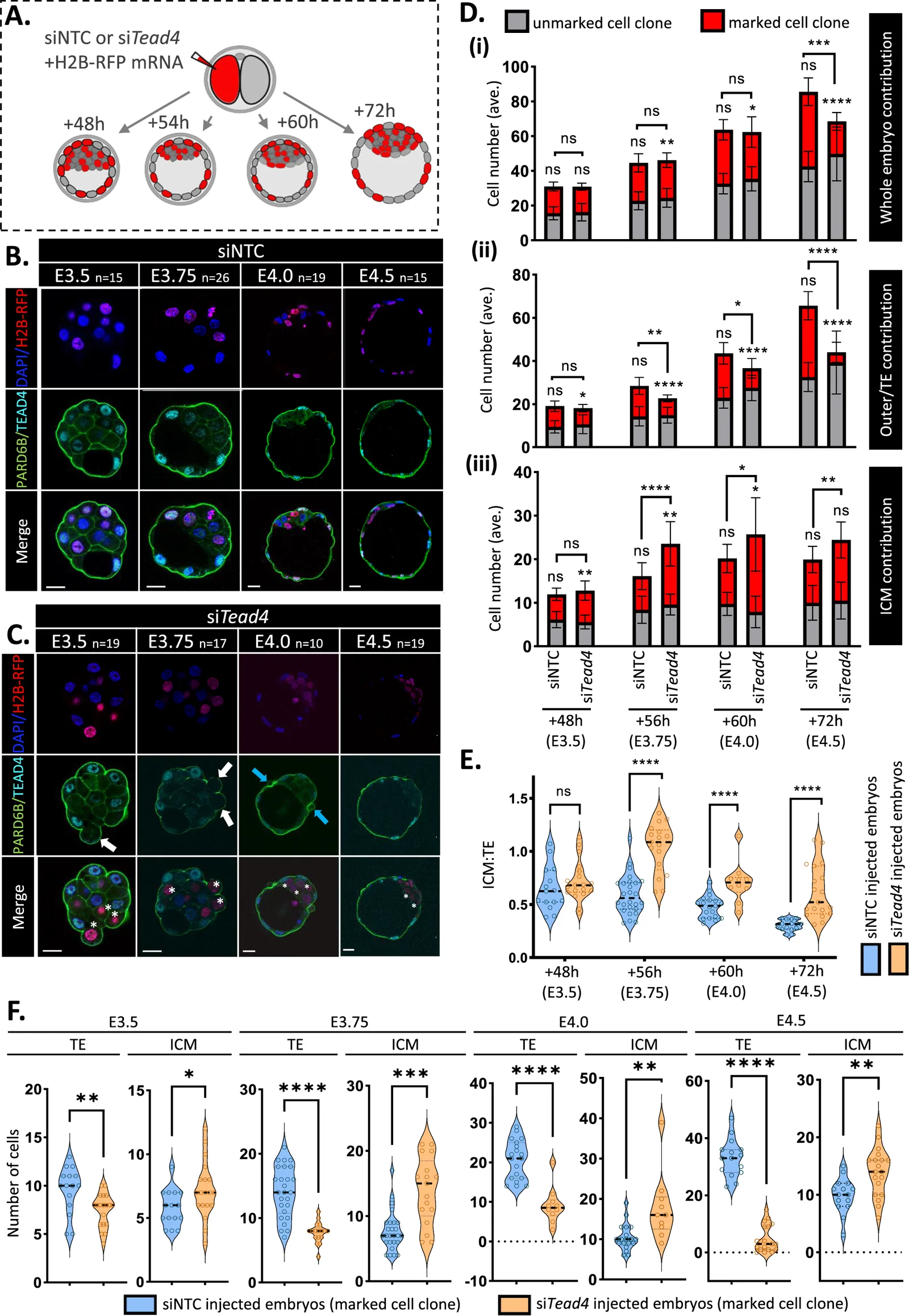
Clonal *Tead4* KD induces atypical cell allocation events during blastocyst maturation. (A) Schematic of siRNA microinjection procedure generating staged blastocysts containing either control (siNTC) or *Tead4* KD (si*Tead4*) marked clones—comprising 50% of all cells; allowing assessment of clone contributions to the outer/TE or ICM positions. (B) Example confocal micrographs (single merged z-sections) of IF-stained siNTC-injected blastocysts (targeted clones—red nuclei); PARD6B (green) and TEAD4 (cyan), with DAPI-DNA counterstain (blue). Scale bar, 20 µm; number of embryos per group (*n*) indicated (see also supplementary material, figure S1 for greyscale images). (C) Similar images as in panel B, but for si*Tead4*-injected blastocysts. Clonal *Tead4* KD cells are marked with asterisks. Morphological abnormalities, such as apical domain blebs and distortions, are indicated with white arrows (E3.5 and E3.75). Vesicle-like structures containing PARD6B are marked with blue arrows (E4.0). (D) Quantification of clone contributions to (i) total, (ii) outer/TE and (iii) ICM cell numbers across different blastocyst stages: unmarked (grey) versus marked (red) clones within siNTC and si*Tead4* blastocysts (data are also presented as violin plots and illustrative concentric pie-charts of percentage clone composition in outer/TE and ICM lineages; supplementary material, figure S1). (i) Whole-embryo contribution: no significant differences (*n*s, *p*>0.05) between unmarked/marked cells were observed within siNTC embryos across all stages. No significant differences between unmarked/marked cells were observed within si*Tead4* embryos at E3.5. Significant differences (*p < 0.05, **p < 0.01, ****p < 0.0001) were observed between unmarked/marked cells within si*Tead4* embryos at E3.75–E4.5. No significant difference in whole-embryo cell number was observed between siNTC and si*Tead4* embryos at E3.5–E4.0. A significant difference (***p < 0.001) was observed between siNTC and si*Tead4* embryos at E4.5. (ii) Outer/TE contribution: no significant differences (*n*s, *p* > 0.05) between unmarked/marked TE cells were observed within siNTC embryos across all stages. Significant differences (*p < 0.05, ****p < 0.0001) were observed between unmarked/marked cells within si*Tead4* TE at all stages. No significant difference between siNTC and si*Tead4* TE cell numbers was observed at E3.5. Significant differences (*p < 0.05, **p < 0.01, ****p < 0.0001) were observed between siNTC and si*Tead4* TE cell numbers at E3.75–E4.5. (iii) Inner/ICM contribution: no significant differences (*n*s, *p* > 0.05) between unmarked/marked ICM cells were observed within siNTC embryos across all stages. Significant differences (*p < 0.05, **p < 0.01) were observed between unmarked/marked ICM cells within si*Tead4* embryos at all stages. No significant difference between siNTC and si*Tead4* ICM cell numbers was observed at E3.5. Significant differences (*p < 0.05, **p < 0.01, ****p < 0.0001) were observed between siNTC and si*Tead4* ICM cell numbers at E3.75–E4.5. Within-group comparisons (marked versus unmarked clones within the same embryo) were performed using paired *t-*tests or Wilcoxon signed-rank tests; between-group comparisons (siNTC versus si*Tead4* embryos) were performed using unpaired *t-*tests or Mann–Whitney *U*-tests. Each statistical test was selected according to the parameter comparison and data distribution. Error bars represent the s.d. of within-group datasets. (E) ICM: outer/TE cell number ratios in blastocysts containing marked siNTC (blue) and si*Tead4* (orange) clones at specified stages. No significant difference (*n*s, *p* > 0.05) between the groups was observed at E3.5. Significant differences (****p < 0.0001) between the groups were observed at E3.75–E4.5. Between-group comparisons were performed using unpaired *t-*tests, selected according to data distribution. Dashed lines denote median values. (F) Comparison of outer/TE and ICM cell number contributions of only the marked siNTC (blue) versus si*Tead4* (orange) clones, at the indicated stages. Significant differences (*p < 0.05, **p < 0.01, ***p < 0.001, ****p < 0.0001) were observed between siNTC and si*Tead4* embryos in both lineages at all stages. Between-group comparisons were performed using unpaired *t-*tests or Mann–Whitney *U*-tests, selected according to data distribution. Dashed lines denote median values. All clonal allocation raw data are summarized in the supplementary material, table S1a–e. Full details of the test selection are provided in §4.8. Exact *p-*values and the specific test applied for each individual comparison are provided in Excel workbook in the supplementary material.

We then compared the development of embryos microinjected with si*Tead4* versus siNTC (see supplementary material, table S1a–e for detailed cell counts). In siNTC controls, blastocyst total cell numbers increased steadily from E3.5 to E4.5, with marked and unmarked clones contributing equally, indicating no adverse developmental impact. In si*Tead4* embryos, total cell numbers were comparable to controls from E3.5 to E4.0, but by E3.75, marked *Tead4* KD clone numbers were slightly reduced, with evident compensation from unmarked clones (extending to the E4.0 stage). At E4.5, si*Tead4* blastocysts had significantly fewer total cells on average (68.5 ± 3.28 versus 85.5 ± 1.7), mainly owing to fewer marked clone cells, despite partial compensation from unmarked clones (figure 1D; supplementary material, figure S1A,B). Overall, embryo cell numbers were similar until E4.0, after which si*Tead4* embryos had fewer overall cells, exclusively in the marked clone.

We next assessed marked and unmarked clone contributions to outer and ICM positions (figure 1D–F; supplementary material, figure S1A–C). In siNTC controls, there was no significant difference between clones in either lineage at any stage, indicating unbiased contribution and normal development (see also clonal TE/ICM percentage composition; supplementary material, figure S1C). Additionally, the ICM : TE cell ratio decreased appropriately over time, demonstrating normal blastocyst development after siNTC microinjection. By contrast, si*Tead4* clones contributed only 12% of cells to a significantly smaller TE by E4.5, consistent with the characterized role of *Tead4* in TE development [21,22]. Conversely, si*Tead4*-microinjected blastocysts had larger ICMs, with over half (57%) of the cells originating from the marked clone, and significantly increased marked ICM cell numbers compared with marked ICM clones in siNTC controls. These findings confirm a strongly biased ICM contribution from *Tead4* KD clones, even in embryos with significantly fewer marked clones overall, resulting in late (E4.5) blastocysts with predictably reduced TE but atypically expanded ICM.

At E3.75 and E4.0, where overall cell numbers were similar between si*Tead4* and siNTC embryos, si*Tead4* clones made up a larger proportion of the total ICM at 59 and 68%, compared with 48 and 51%, respectively, for the siNTC clone controls. This led to overall significantly smaller TE and larger ICM sizes. Accordingly, the reduced TE contribution was not owing to a smaller clone size (as seen at E4.5) but indicated a genuine positional bias caused by *Tead4* KD. Notably, at E3.5, no significant differences were observed in overall TE or ICM cell numbers. However, within just 6 hours (from E3.5 to E3.75), si*Tead4* embryos exhibited a marked shift in positional allocation, averaging 6.32 fewer TE cells and 6.25 more marked ICM cells. This trend continued at E4.0, with an additional 7.38 marked ICM cells. This suggests a post-cell division mechanism whereby outer blastomeres at the E3.5 stage were atypically redistributed internally after the initial formation of primary and secondary ICM founders. Morphologically, the outer cells of si*Tead4* clones displayed distinctive abnormalities in their apical membranes, which we termed apical blebs (figure 1B,C; supplementary material, figure S1A,B). These abnormalities were particularly evident at E3.5 and E3.75, coinciding with the observed large cell positioning shifts. Additionally, vesicle-like structures containing the apical polarity factor PARD6B were found on outer embryo surfaces (figure 1B,C; supplementary material, figure S1A,B), potentially representing apical bleb abscission events; theoretically resulting from increased contractility, which could facilitate the reallocation of marked *Tead4* KD outer cells to the ICM.

At E3.5, si*Tead4*-treated blastocysts show no significant difference in total TE or ICM cell numbers compared with controls, but the microinjected clone did contribute less to the TE and more to the ICM, indicating early clonal allocation differences starting before the 32-cell stage; likely during the 16- to 32-cell transition. These data also reveal complete compensatory regulation by the unmarked clone, balancing overall TE and ICM cell allocation in E3.5 stage si*Tead4*-treated embryos. Additionally, at E4.0, unmarked ICM cells in si*Tead4*-treated blastocysts were actually reduced in number compared with those at E3.75 (and the equivalent unmarked cells of siNTC embryos), suggesting further compensatory mechanisms attempting to regulate overall ICM size, following the atypical influx of marked clones. Similar reductions in marked si*Tead4* ICM cells between E4.0 and E4.5 confirm such regulative mechanisms (exemplified by reducing ICM: TE/outer cell ratios from the E3.75 peak; figure 1E).

TEAD4–YAP1 complexes in outer cells activate TE-specific genes like *Cdx2* through polarity-dependent suppression of Hippo signalling [21,22,27]. We confirmed that global si*Tead4* KD (achieved by microinjecting both two-cell stage blastomeres) reduces the expression of TE marker mRNAs, such as *Gata3* [31], *Tfap2c* [32] and *Cdx2* [33] (supplementary material, figure S1D–F; table S2), by the E3.5 blastocyst stage. To determine if such genes also contribute to the atypical outer cell-to-ICM cell allocations, we clonally disrupted *Tfap2c* and *Cdx2* using dsRNA or CRISPR-Cas9 via similar microinjection methods, then examined blastocyst development (supplementary material, figures S2 and S3; tables S3a–S4b). Despite successful, clone-specific loss of detectable TFAP2C and CDX2 protein expression, we found no evidence of reduced outer cell contribution or increased ICM contribution. Additionally, there were no changes in the morphology of marked outer clone apical domains. These results suggest that, although TEAD4 regulates *Tfap2c* and *Cdx2* expression, neither target gene alone mediates the specific regulation of outer cell positioning attributable to *Tead4*. Instead, TEAD4 likely influences this process through its broader transcriptional network.

Overall, these data show that clonal si*Tead4* KD leads to late blastocysts (E4.5) with significantly more ICM and fewer TE cells. This occurs because marked clones are preferentially allocated to the ICM starting as early as the 32-cell stage (E3.5), despite compensatory masking in unmarked clones maintaining an appropriate ICM : TE/outer cell ratio. However, between E3.5 and E3.75, marked clones robustly and atypically populate the ICM, likely through direct outer cell-to-ICM re-allocation/internalization events, supported by morphological apical domain distortions in outer cells. This abnormal allocation persists into the late (E4.5) blastocyst stage, with varying compensation within unmarked and marked ICM and outer-residing clones. However, similar targeted clonal dysregulation of confirmed TEAD4 target TE-specific genes, *Tfap2c* and *Cdx2*, is unable to elicit similar allocation phenotypes. Thus, TEAD4 plays a key role in maintaining relative cell spatial positioning and ensuring TE cells occupy outer lineage-appropriate positions within the maturing blastocyst.

### 2.2. *Tead4*-specific siRNA knockdown clones internalize via typical asymmetric cell divisions and apical domain constriction, and unconventional mechanisms associated with apical bleb formation and abscission

Having identified an early blastocyst window (E3.5–E4.0), characterized by atypical outer cell-to-ICM allocation, apical bleb formation and PARD6B-containing surface vesicles in *siTead4* KD clones within fixed embryos, we employed fluorescent light-sheet microscopy four-dimensional live-cell imaging to monitor the dynamics by which these phenotypes emerge, and with greater spatiotemporal resolution. We co-microinjected single blastomeres from transgenic two-cell stage embryos expressing a fluorescent plasma membrane-localized Tomato reporter (mT [34]) with either siNTC or si*Tead4* and mRNA encoding histone H2B-Venus (as an injection/lineage marker). Microinjected embryos were cultured to 89 hours post-hCG and then imaged at intervals of 10 minutes for the following 16.5 hours to assess the dynamic behaviour of H2B-Venus-marked control and *Tead4* KD clones, compared with the unmarked clones (marked only by mT) from the same embryos (figure 2A).

**Figure 2.**
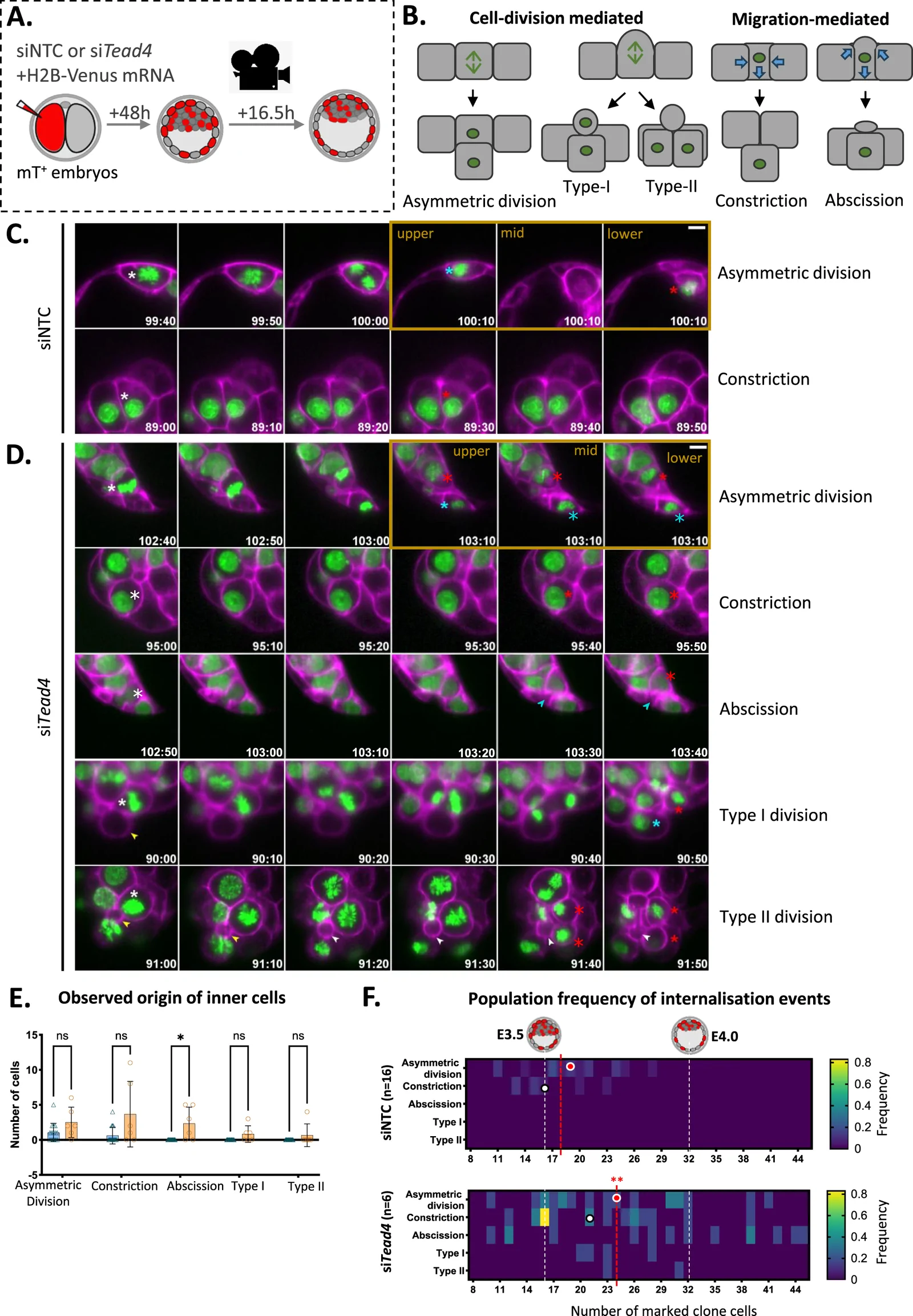
Four-dimensional light-sheet live-cell embryo imaging of atypical blastocyst internalization of marked *Tead4* KD cell clones. (A) Experimental schematic of two-cell stage microinjections of transgenic membrane-Tomato expressing (mT^+^) two-cell embryos used to generate early 32-cell stage blastocysts (after 48 hours of culture) containing either histone H2B-Venus marked control (siNTC) or *Tead4* KD (si*Tead4*) clones (comprising 50% of all cells), subject to 16.5 hours of light-sheet live-cell imaging (10-minute intervals); allowing dynamic assessment of individual clone contributions to the outer/TE or ICM positions. (B) Schematic representations of the cell internalization mechanisms observed for marked (H2B-Venus+) clones during blastocyst maturation. (C) Examples of cell internalization events observed in embryos containing marked siNTC clones (i.e. asymmetric cell divisions and apical domain constriction), as successive time-lapse imaging frames (timings reflect hh : mm post-hCG superovulation injection and scale bar, 10 µm). The cell marked with a white asterisk displays the phenotype described next to the image; cyan and red asterisks display derived outer and inner cells, respectively. For asymmetric cell divisions, the last three frames represent the same time point in three consecutive z-sections (highlighted by the orange box). Pseudo-colour scheme: magenta—transgenic embryo mT reporter (as a single z-plane) and green—histone H2B-Venus microinjection marker (as a maximum projection from several planes located above and below the selected mT plane). Micrographs taken from the illustrative blastocyst embryo depicted in movie S1 in the supplementary material. (D) Examples of cell internalization events observed in embryos containing marked si*Tead4* clones (i.e. asymmetric cell divisions, apical domain constriction and the additional atypical blastocyst internalization mechanisms are summarized in panel B), as successive time-lapse imaging frames (timings reflect hh : mm post-hCG superovulation injection and scale bar, 10 µm). Cells marked with a white asterisk exhibit the phenotype described next to the image, and cyan and red asterisks denote outer and inner cells, respectively. Blue arrowheads denote remnants of the apical domain following apical abscission (after cell internalization independent of cell division), yellow arrowheads indicate formation of apical blebs (associated with types I and II cell divisions) and white arrowheads highlight the formation of surface vesicles (devoid of segregated chromosomes, after type II cell divisions). For asymmetric cell divisions, the last three frames represent the same time point in three consecutive z-sections (highlighted by the orange box). Pseudo-colour scheme: magenta—transgenic embryo mT reporter (as a single z-plane) and green—histone H2B-Venus microinjection marker (as a maximum projection of several planes located above and below the selected mT plane). Micrographs taken from the illustrative blastocyst embryos are depicted in movie S1 in the supplementary material. (E) Quantification of the total number of marked ICM cells at the end of the imaging period, which were derived from an internalized outer/TE cell, as illustrated in panels C and D. No significant differences (*n*s, *p* > 0.05) were observed in the final population of siNTC and si*Tead4* ICM cells, which arose from asymmetric divisions, apical constriction, type-I and type-II internalizations. A significant difference (*p < 0.05) was observed in ICM cells arising from apical abscission internalization. Between-group comparisons were performed using Mann–Whitney *U*-tests, selected according to data distribution. To ensure rigorous testing between multiple groups, a Bonferroni correction was applied to adjust the significance thresholds. Bar heights represent mean values, and error bars denote s.d. (F) Heat map showing the distribution of internalization events at a given developmental stage (delineated by total number of marked clones—representing 50% of the embryo; white-dashed lines denote blastocyst stages equivalent to E3.5 and E4.0). The internalizations represent the population frequencies at each cell stage, normalized to the number of blastocysts imaged. Combined, internalization events of marked clones (irrespective of origin) occur significantly later, on average, in si*Tead4* embryos than in siNTC embryos (mean timing denoted by red-dashed lines, **p < 0.005). Internalization events driven by asymmetric division and constriction occur in both siNTC and si*Tead4* embryos, allowing for direct statistical comparison; the mean cell number of internalizations is denoted by red and white dots, respectively. No significant difference in the distribution was observed between either the group (*n*s, *p* > 0.05), following Bonferroni correction of *p-*values. Between-group comparisons were performed using unpaired *t-*tests (combined internalization and asymmetric internalization distributions) and the Mann–Whitney *U*-test (constriction internalization distribution), selected according to data normality. Detailed lineage trees for all analysed embryos are provided in the supplementary material, figures S4 and S5. Note that the details of the ultimate origin of injected/marked cells at the end of the movie and the observed frequencies of internalization events are contained in table S5a–d in the supplementary material. Full details of test selection are provided in §4.8. Exact *p-*values and the specific test applied for each individual comparison are provided in Excel workbook in the supplementary material.

We found that the marked clones of si*Tead4*-treated blastocysts are internalized through an increased occurrence of classically described internalization mechanisms, including the emergence of other undescribed processes. Broadly, these can be divided into division-mediated and migration-mediated events. Internalizations were linked to an increase in classical asymmetric divisions, in which an outer cell divides parallel to the apical–basal axis (a perpendicular division plane) to generate one inner and one outer daughter cell (as described for the generation of both primary and secondary ICM founder cells, prior to the 32-cell stage [7]). We also observed atypical division-mediated internalizations in cells with apical blebs that appear to alter the axis of cell division, leading to what we termed either ‘type I’ or ‘type II’ divisions. In type I divisions, cells divide asymmetrically, with one daughter retaining a surface position and the apical bleb membrane while the other cell internalizes. In type II divisions, the division axis is perpendicular to the apical–basal axis, but both daughters lose surface contact and internalize to the ICM, leaving a surface vesicular apical bleb remnant. Migration-mediated internalizations again include the previously described mechanisms of conventional constriction of a small apical domain, whereby the cell internalizes by progressively reducing its surface contact [11,12,18,35], and unusual apical abscission events. The latter involves the complete resection of the apical domain from non-dividing outer cells, which, post-abscission, yields an inner cell and a vesicular remnant on the blastocyst surface (figure 2B,D).

We used the light-sheet imaging data to construct annotated lineage trees for each siNTC- and si*Tead4*-treated imaged blastocyst, detailing the incidences of each category of internalization event and cellular apoptosis (supplementary material, figures S4 and S5). To understand how clonal si*Tead4*-KD affects the composition of the final ICM population, we determined the proportion of ICM cells derived from internalization, the mechanism by which each founding ICM cell was generated, and the developmental timing of these events. Because a single internalization event can generate multiple daughter cells (for example, after subsequent cell division within the ICM or can be associated with apoptosis), the final composition of the ICM population is not directly comparable to the number of internalization events. Therefore, we separately identified the mechanistic origins of the final ICM population (figure 2E) and the timing of all internalization events of marked clones (figure 2F). With respect to the mechanistic origin of the ICM populations, si*Tead4*-treated embryos show a general increase across all internalization mechanistic origins observed, relative to siNTC controls. Cells derived from atypical apical abscissions were significantly increased. Cells arising from classical asymmetric division and apical domain constriction, including atypical type I and type II division-associated internalization (with the latter observed twice in one si*Tead4*-treated embryo), exhibited similar trends, although these did not reach statistical significance thresholds (figure 2E). In terms of the individual internalization events, in total, we recorded 23 internalization events in siNTC embryos across 16 embryos and 58 events across si*Tead4* embryos across six embryos (****p < 0.0001; supplementary material, table S5b). Separated by mechanism, this comprised 16 asymmetric division events in siNTC versus 19 in si*Tead4* (mean values of 1.00 versus 3.17 events per embryo, respectively; *n*s, *p* > 0.05—supplementary material, table S5b) and seven constriction events in siNTC versus 19 in si*Tead4* (means of 0.44 versus 3.17 events per embryo, *p < 0.05; supplementary material, table S5b). Abscission (14 events), type I division (four events) and type II division (two events) were observed exclusively in si*Tead4* embryos (supplementary material, table S5b). To characterize the timing of these internalization events, we mapped the internalization frequencies against the number of marked cells in both conditions (figure 2F; supplementary material, table S5c). Combined, internalization events across all mechanisms observed occurred, on average, significantly later and persisted longer into blastocyst maturation in si*Tead4*-treated embryos than in siNTC embryos (**p < 0.001; supplementary material, table S5d). Asymmetric division and constriction were the only mechanisms represented in both groups, allowing direct statistical comparison of internalization timing; however, no significant difference in timing was observed between groups for either mechanism (supplementary material, table S5d).

Our frequency observations also aligned with the fixed-embryo data showing that the marked *Tead4* KD clone contributes statistically more inner cells than its unmarked counterpart by the early blastocyst stage (E3.5), despite overall compensatory regulation from the unmarked clone in terms of total outer and ICM cell number (figure 1; supplementary material, figure S1). Interestingly, we did observe unanticipated internalization of marked siNTC clones during the post-32-cell (E3.5) blastocyst maturation phase (figure 2E,F). While these data indicate that outer-to-inner cell transitions are possible beyond the 32-cell stage under control conditions, their frequency was considerably reduced and exclusively restricted to the classically described internalization mechanisms (i.e. asymmetric cell division and apical domain constriction). Moreover, post-32-cell-stage internalization of marked siNTC clones was limited to the earlier phase of blastocyst maturation, whereas similar mechanisms, including those associated with apical domain abscission and atypical cell divisions, persisted longer and at a higher frequency in marked *Tead4* KD clones (figure 2F).

Overall, these data, together with those from fixed blastocyst samples (figure 1; supplementary material, figure S1), show that marked *Tead4* KD clones become abnormally internalized into the ICM, starting around the 32-cell stage (E3.5) and continuing throughout blastocyst maturation, contributing to an increased number of ICM cells. The mechanisms involve: increased incidence of conventional asymmetric cell division and apical domain constriction, and unusual processes related to apical domain abscission and defective cell divisions associated with apical bleb formation. Collectively, these findings support a homeostatic role for *Tead4* in ensuring the correct relative spatial positioning of TE-specified cells within the blastocyst.

### 2.3. Clonal *Tead4* knockdown reduces, but does not ablate, outer cell apical polarity and disrupts YAP1 localization

From the 16-cell stage, outer cell TE specification is tightly controlled by intracellular apical-basolateral polarity, which suppresses Hippo signalling through sequestration of AMOT at the apical domain. This ensures nuclear TEAD4–YAP1 complex formation and TE-related gene expression [21,22,36]. Indeed, chemical inhibition of ROCK1/2 (i.e. Rho-associated coiled-coil-containing protein kinases) completely disrupts outer cell polarity, characterized by uniform plasma membrane distribution of polarity factors, ectopic AMOT at basolateral sites, activation of Hippo signalling and loss of CDX2 expression and TE-specification [37,38]. Apical polarity establishment begins at the late eight-cell stage, preceding spatial segregation of blastomeres, in two steps: first, formation of a cortical actomyosin network and second, polarity protein recruitment. This process is asynchronous, dependent on redundant *Tead4* and *Tfap2c* function, including active RHOA protein, with early polarizing blastomeres more likely to contribute to the TE lineage [16,17,39]. However, in addition to specification, maintaining polarity is essential for correct outer TE cell positioning. Time-lapse imaging shows that loss of polarity leads to ICM internalization of outer cells [18], and clonally targeting apical polarity proteins like PARD3 and PRKCZ cause outer cells to similarly re-localize [19]. Hence, establishing apical-basolateral polarity promotes TE gene expression but also ensures proper spatial placement of outer TE cells, a concept we termed the ‘polarity-dependent cell-positioning model’ [28]. To investigate this further, we examined whether impaired polarity and ectopic Hippo signalling occur in blastocysts containing *Tead4* KD clones.

Our analysis of fixed blastocysts revealed that outer cells in both siNTC and si*Tead4* marked clones displayed apical membranes appropriately associated with PARD6B expression, including within apical blebs or formed surface vesicles associated with outer cell-to-ICM reallocation (figures 1 and 2; supplementary material, figure S1). This shows that apical-basolateral polarity is established and remains intact in *Tead4* KD outer clones (although it does not exclude quantitative differences in the extent of such polarity). Indeed, similar PARD6B expression was consistently observed in E3.5 blastocysts, also presenting with apical blebs and vesicles, in *Tead4* KD clones independently co-stained for PARD6B and YAP1 (figure 3A; supplementary material, table S6b). However, in such blastocysts, whole-embryo PARD6B levels were significantly lower in those containing marked si*Tead4* clones compared with siNTC controls (figure 3B; supplementary material, table S6b). Analysis of single outer cells showed this reduction was specific to the si*Tead4* clone, which had significantly lower apical PARD6B expression than unmarked outer cells within the same embryo and compared with both siNTC control outer cell clones (figure 3C). These findings align with previous data showing *Pard6b* mRNA levels decrease (to approx. 80%) in E3.5 blastocysts derived from *Tead4* shRNA-injected zygotes [29] and to approximately 50% from zygotes targeted by efficient *Tfap2c* siRNA KD (associated with reduced apical PARD6B protein, albeit at the earlier morula stage [32,40]). We hypothesize that the si*Tead4*-induced decreases in *Tfap2c* mRNA levels (to approx. 40%; supplementary material, figure S1) partly explain the significant, but incomplete, reduction in apical PARD6B in marked outer clones. In summary, si*Tead4*-marked outer cell clones, reallocating to the ICM, show significantly reduced PARD6B levels, indicating diminished but not ablated apical polarity, most likely associated with TEAD4-mediated regulatory gene expression feedback.

**Figure 3.**
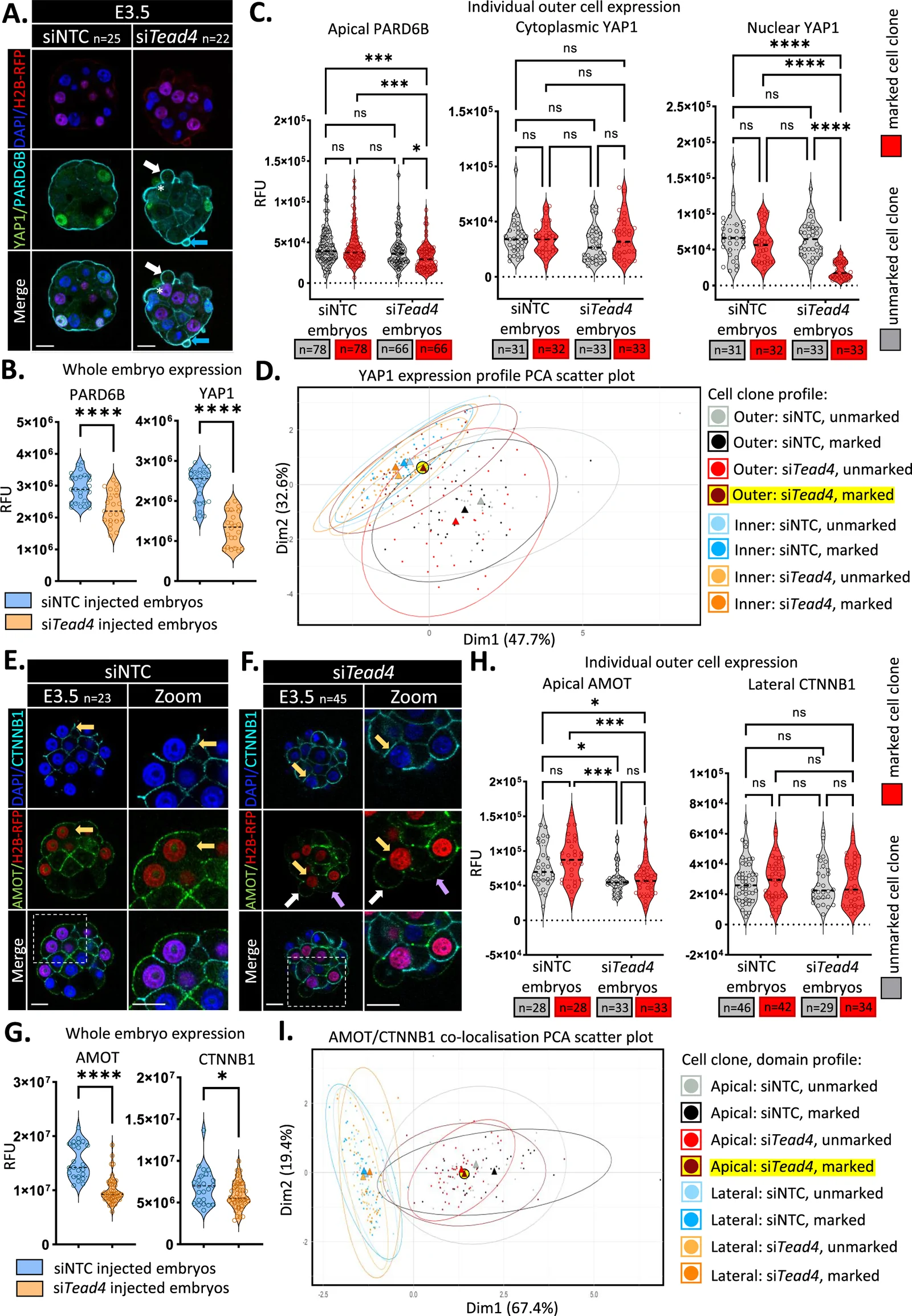
Clonal *Tead4* KD impairs, but does not ablate, outer cell apical domain polarity and affects Hippo signalling components. (A) Example single z-section confocal micrographs of E3.5 blastocysts containing marked (red nuclei) clones injected with siNTC (left) or siTead4 (right) constructs. Embryos are IF-stained for PARD6B (cyan; an apical polarity protein) and YAP1 (green; a Hippo pathway effector and TEAD4 co-factor), including DAPI DNA stain (blue). Scale bar, 20 µm. The number of embryos per group (*n*) is indicated. Asterisks highlight atypical cytoplasmic YAP1 localization in outer cells, instead of the usual nuclear localization (associated with Hippo signalling repression), of siTead4 clones; similarly characterized by apical blebs and surface vesicle-like structures, indicated by white and blue arrows (figure 1). (B) Comparison of PARD6B and YAP1 expression levels (normalized relative fluorescence units, RFU) across whole embryos at E3.5, including unmarked and marked clones, in control siNTC (blue) and si*Tead4* (orange) embryo groups; as illustrated in panel A. Significant differences (****p < 0.0001) were observed between groups. RFU measurements represent independent data points and are pooled according to their condition (siNTC and si*Tead4* embryos). Between-group comparisons were performed using Mann–Whitney *U*-tests, selected according to data distribution. Dashed lines denote median values. (C) Quantitative analysis of individual outer cell expression levels (RFU) for apical membrane-associated PARD6B, including cytoplasmic and nuclear localized YAP1, in siNTC and si*Tead4* blastocysts. Data are separated for unmarked (grey) and marked (red) clone populations. No significant differences (*n*s, *p* > 0.05) were observed in cytoplasmic YAP1 expression between all cells. No significant differences were observed in apical PARD6B and nuclear YAP1 expression levels between siNTC unmarked/marked cells and *siTead4* unmarked cells. Significant differences (*p < 0.05, ***p < 0.001, ****p < 0.0001) were observed in apical PARD6B and nuclear YAP1 expression levels between marked si*Tead4* cells and all other cells. RFU measurements represent independent data points and are pooled according to their condition (siNTC/si*Tead4* and unmarked/marked embryos). Between-group comparisons across the four clone populations were performed using Mann–Whitney *U*-tests or unpaired *t-*tests, selected according to data distribution. Dashed lines denote median values (see supplementary material, figure S6 for similar data on ICM cell YAP1 expression). (D) Principal Component Analysis (PCA) of subcellular YAP1 expression levels, comparing unmarked and marked clones in siNTC and si*Tead4* blastocysts (supplementary material, figure S6). Each point represents an individual cell, with ellipses denoting 95% confidence intervals. Mean cluster centres are marked with triangles—outer marked si*Tead4* KD clones highlighted in yellow. (E,F) Similar to panel A, but reporting AMOT (green) and CTNNB1 (cyan) IF-staining in (E) siNTC-treated and (F) si*Tead4-*treated blastocysts, including magnified insets. White and purple arrows indicate weak but correctly localized apical AMOT in marked and unmarked outer cells, respectively. Yellow arrows denote the absence of any co-localization of AMOT and CTNNB1 on marked outer cell lateral membranes in either embryo group. (G) Comparison of whole-embryo expression levels of AMOT and CTNNB1, derived from the same embryo groups illustrated in panels E and F. Significant differences (*p < 0.05, ****p < 0.0001) were identified between both groups. RFU measurements represent independent data points and are pooled according to their condition (siNTC and si*Tead4* embryos). Between-group differences were assessed using Mann–Whitney *U* tests (AMOT) or unpaired *t-*tests (CTNNB1), selected according to data distribution. Dashed lines denote median values. (H) Similar to panel C, quantification of single outer cell expression levels (RFU) for apical membrane-associated AMOT and lateral membrane-localized CTNNB1 in siNTC and si*Tead4* blastocysts (see supplementary material, figure S7 for similar ICM expression data). No significant differences (*n*s, *p* > 0.05) were identified in lateral CTNNB1 expression levels in all groups and in apical AMOT expression levels between unmarked/marked cells within both siNTC and si*Tead4* embryo groups. Significant differences (*p < 0.05, ***p < 0.001) were identified in apical AMOT expression between siNTC and si*Tead4* embryos across both unmarked/marked clones. RFU measurements represent independent data points and are pooled according to their condition (siNTC/si*Tead4* and unmarked/marked embryos/clones). Between-group comparisons were performed using Mann–Whitney *U*-tests or unpaired *t-*tests, selected according to data distribution. Median values are denoted by dashed lines. (I) PCA plot similar to panel D, reflecting co-localization and expression levels of AMOT and CTNNB1 at outer cell apical and lateral membranes (supplementary material, figure S7)—outer marked si*Tead4* KD clone, apical domain analyses, highlighted in yellow. All quantified protein expression values (RFU) data are summarized in the supplementary material, tables S6a,b and S7a,b. Full details of the test selection are provided in the Statistical analysis section of the Material and methods. Exact *p-*values and the specific test applied for each individual comparison are provided in Excel workbook in the supplementary material.

Alongside PARD6B, we examined the expression of the polarity-sensitive Hippo signalling effector YAP1. Unlike both outer siNTC control clones and the unmarked outer clones of si*Tead4* embryos, the marked *Tead4* KD outer clones did not display typical nuclear YAP1 localization (which normally indicates intact apical-basolateral polarity and suppressed Hippo signalling [21,22]). Instead, YAP1 was predominantly cytoplasmic, resembling all assayed apolar inner cell populations (figure 3A; supplementary material, table S6a). This implies that, in addition to reduced polarity, such clones may exhibit some mechanistic impairment in Hippo pathway suppression. Whole-embryo YAP1 expression was also significantly reduced after clonal si*Tead4* treatment, but specifically within the nuclei of marked outer clones, as cytoplasmic levels were unchanged (figure 3B,C; see supplementary material, figure S6A for inner cell data). Indeed, the average nuclear : cytoplasmic YAP1 expression ratio robustly and significantly decreased in such marked outer clones, more closely resembling inner cell values (supplementary material, figure S6B). Principal Component Analysis (PCA) of YAP1 expression/localization further confirmed this shift, as marked si*Tead4* outer clones moved away from the group of control outer clones and towards the tightly clustered inner cell profiles (figure 3D; supplementary material, figure S6D–F). These findings demonstrate that *Tead4* KD causes a cell-autonomous reduction in nuclear YAP1 in outer cells, supporting the existence of feedback mechanisms that sustain polarity-dependent suppression of Hippo signalling and TE differentiation. The altered YAP1 localization closely mimics inner cell populations, suggesting that *Tead4* KD in clones undergoing outer cell-to-ICM reallocation may lead to aberrant Hippo pathway activation.

To investigate whether *Tead4* KD induces ectopic outer cell activation of the Hippo pathway, we performed IF staining of clonal siNTC- and si*Tead4*-injected E3.5 blastocysts for AMOT and the basolaterally localized adherens cell junction (AJ) marker CTNNB1 (β-Catenin [41]). From the 16-cell morula stage, AMOT activates Hippo signalling by localizing to inner cell AJs and promoting LATS1/2-dependent phosphorylation of YAP1, leading to its cytoplasmic retention; an association blocked in outer cells owing to AMOT sequestration at the contactless and polarized apical domain [21,22]. Hence, we assayed for AMOT mislocalization to the lateral domains of outer cells, marked by CTNNB1 expression, which would indicate aberrant Hippo pathway activation (figure 3E). In control siNTC blastocysts, AMOT was normally confined to the apical domain of outer cells, while CTNNB1 was appropriately basolateral and inner cells showed co-localization consistent with active Hippo signalling. In si*Tead4* embryos, despite the presence of marked outer clone-specific apical blebs (marking ICM reallocation), no evidence of abnormal AMOT or CTNNB1 lateral domain co-localization was observed in either marked or unmarked clones (note that outer cell basal membrane localization cannot be unambiguously assigned owing to neighbouring inner cell plasma-membrane associated signal). Indeed, AMOT was correctly localized to outer cell apical membranes (as was CTNNB1 to all cell-contacted membranes) and the plasma membranes of inner cells. However, AMOT expression levels in general appeared lower than in siNTC controls (figure 3E,F). Indeed, whole-embryo quantification analyses confirmed robustly reduced AMOT and modestly reduced CTNNB1 in si*Tead4* blastocysts (figure 3G; supplementary material, table S7a), and on the single-cell level, *Tead4* KD caused significant reductions in apical AMOT in both marked and unmarked outer clones, with no significant changes in lateral CTNNB1 expression (figure 3H; supplementary material, table S7b). Similar clone-independent reductions were also seen in inner cell clones, though CTNNB1 expression was unaffected (supplementary material, figure S7A). These analyses demonstrate clonal *Tead4* KD decreases AMOT expression globally, likely through secondary, non-cell-autonomous mechanisms, but has no effect on its subcellular localization (and CTNNB1 expression). Further PCA plots, describing AMOT and CTNNB1 expression levels at outer cell apical and lateral membranes, showed both control siNTC and si*Tead4* KD marked and unmarked clones co-segregated into two main clusters: one with high CTNNB1 and low AMOT levels at lateral membranes (indicating a lack of any *Tead4* KD-induced atypical co-localization), and a more dispersed apical membrane cluster, associated with reduced and clone-independent AMOT levels in si*Tead4* treated blastocysts; note, tight association of mean-centroids versus equivalent siNTC clones (figure 3I; supplementary material, figure S7B–D). Such a membrane-of-origin PCA separation indicates minimal differences in outer cell AMOT and CTNNB1 (co-)localization, associated with clonal *Tead4* KD.

Overall, the outer cell-to-ICM reallocations caused by clonal *Tead4* KD are linked to a significant reduction in apical polarity and lower clone-independent levels of AMOT across the early blastocyst (E3.5). Despite these changes, there is no evidence of AMOT mislocalization to the lateral membranes of outer cells. Therefore, although marked outer si*Tead4* clones display atypical cytoplasmic YAP1 expression, it is highly unlikely that this results from the aberrant activation of the classical-recognized Hippo signalling pathway [21,22]. This strongly suggests that Hippo pathway activity is not the primary driver of the observed allocation phenotypes. Instead, TEAD4 is more likely involved in maintaining the proper spatial positioning of TE cells through a broader regulatory mechanism at the transcriptomic level, although this can positively influence apical polarity and appropriate Hippo signalling feedback, particularly at the level of YAP1 and AMOT protein expression.

### 2.4. Transcriptomic analysis of internalizing *Tead4* knockdown clones identifies dysregulated cytoskeletal gene expression in early-stage (E3.5) blastocysts

To further examine the role of the transcription factor TEAD4 in outer cell apical domain homeostasis and blastomere spatial positioning, we performed RNA-Seq transcriptomic profiling of outer *Tead4* KD clones. Single two-cell stage blastomeres were co-injected with dsRNA targeting *Tead4* (ds*Tead4*) and fluorescent rhodamine-conjugated dextran beads (RDBs—as an injection/lineage marker), and embryos were cultured to the 32-cell (E3.5) stage, concurrent with apical bleb formation and the onset of the observed atypical cellular internalizations. We opted for a ds*Tead4*-mediated rather than si*Tead4*-mediated approach to use the non-genetically encoded RDB injection marker for the transcriptome assays (instead of H2B–RFP mRNA). Based on our experience, *Tead4* KD efficiency was superior in this context when using the previously reported ds*Tead4* construct [27]. After removal of the *zona pellucida*, blastocysts were briefly cultured with yellow-green microspheres (YGMs) that are readily endocytosed by outer cells. Following embryo disaggregation into single cells, four populations were identifiable: (i) inner cells unmarked by RDBs or YGMs (derived from the non-microinjected two-cell -stage blastomere), (ii) inner cells marked by RDBs alone (from ds*Tead4*-microinjected clones), (iii) outer cells marked by YGMs alone (from unmarked outer populations), and (iv) outer cells marked by both RDBs and YGMs (from *Tead4* KD outer clones) (figure 4A). Therefore, we analysed populations (iii) and (iv) by RNA-Seq to compare outer cell transcriptome composition between *Tead4* KD and control unmarked clones from the same blastocysts.

**Figure 4.**
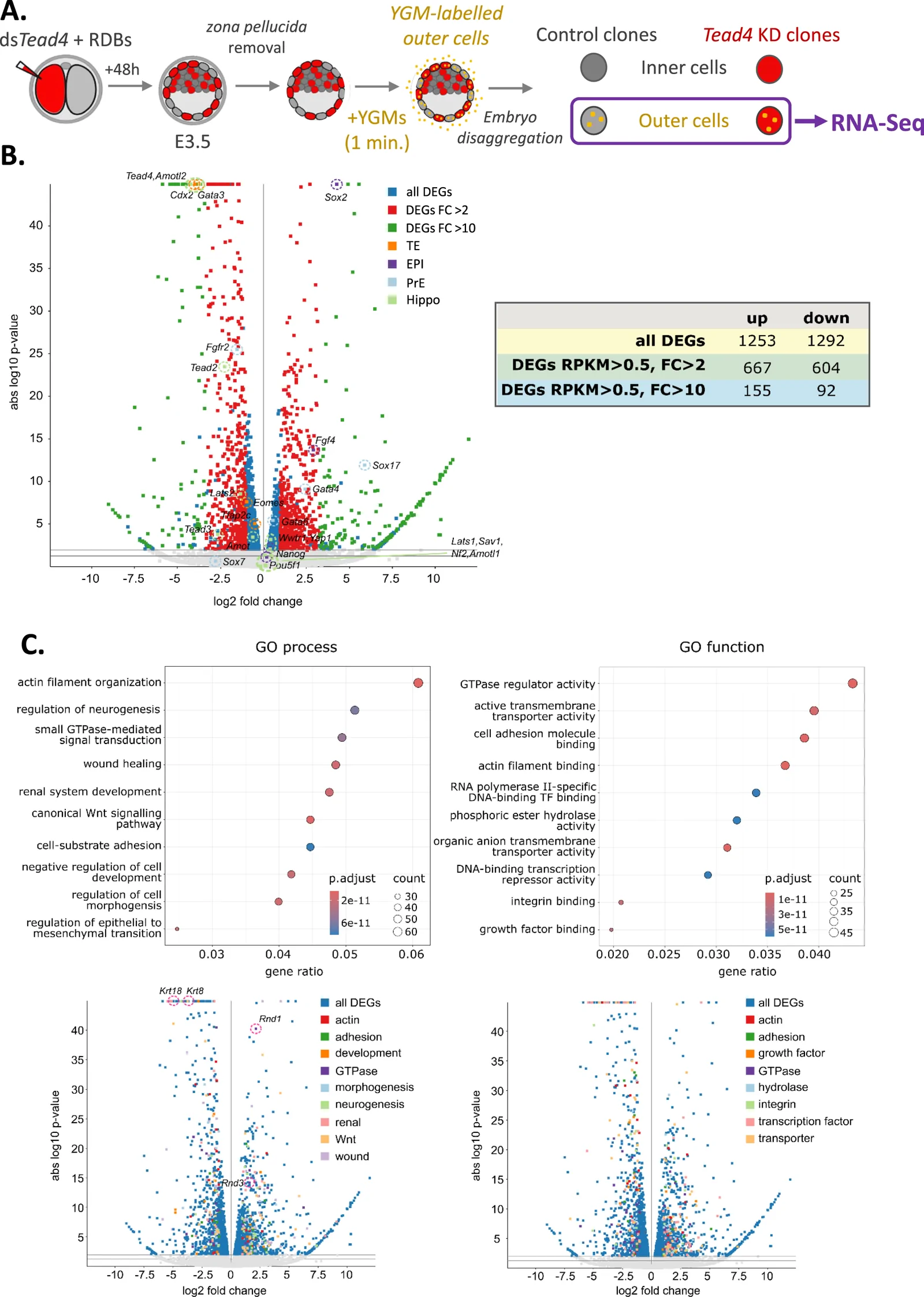
RNA-Seq; *Tead4* KD-mediated dysregulation of cytoskeleton-, actin- and cell adhesion-related gene expression. (A) Schematic of the dsRNA microinjection protocol used to generate E3.5 blastocysts containing *Tead4* KD (ds*Tead4*) clones, marked by co-injection with RDBs, representing 50% of all cells. At E3.5, outer cells were labelled via endocytosis of YGMs, followed by full embryo dissociation into single cells, to enable the identification of inner and outer cell populations from marked and unmarked clones, and preparation of RNA-Seq libraries to compare outer cell transcriptomes. (B) Left: volcano plot showing gene expression differences between RNA-Seq transcriptomes of E3.5 *Tead4* KD (marked) and control (unmarked) outer clone cell populations. Differentially expressed genes (DEGs) are shown in blue, but those with >twofold and >10-fold changes are highlighted in red and green, respectively. Key marker genes for TE, EPI and PrE lineages, as well as Hippo pathway components, are annotated. Right: summary of upregulated and downregulated DEGs, including those with >twofold and >10-fold expression changes, each with RPKM >0.5 in at least one clone. (C) Upper panels: dot plots showing the top 10 enriched gene ontology (GO) terms, classified by process (left) and function (right) for DEGs (>twofold change; RPKM >0.5 in at least one clone) between *Tead4* KD and control outer cells. Lower panels: volcano plots displaying DEG distributions by process- (left; highlighting candidate genes for functional analysis in figure 5; *Krt8*, *Krt18*, *Rnd1* and *Rnd3*) and function- (right) related terms. The simplified GO category legends include specific/merged terms, as follows. Process: actin (actin filament organization), adhesion (cell-substrate adhesion), development (negative regulation of cell development), GTPase (small GTPase-mediated signal transduction), morphogenesis (regulation of cell morphogenesis), neurogenesis (regulation of neurogenesis), renal (renal system development), Wnt (canonical Wnt signalling pathway & regulation of epithelial to mesenchymal transition) and wound (wound healing); function: actin (actin filament binding), adhesion (cell adhesion molecule binding), growth factor (growth factor binding), GTPase (GTPase regulator activity), integrin (integrin binding), transcription factor (RNA polymerase II-specific DNA-binding transcription factor binding and DNA-binding transcription factor repressor activity) and transporter (active transmembrane transporter activity and organic anion transmembrane transporter activity). Lists of all significantly identified DEGs (supplementary material, Excel workbook 1) and their association with significantly enriched function- and process-related GO terms (delineated as those showing >twofold downregulation and upregulation or only downregulation or upregulation alone) are summarized in tables S10–S15 in the supplementary material. Comparisons with enriched DEGs identified in E3.25 stage *Tead4* genetic KOs [42] and genes associated with TEAD4 binding in chromatin immunoprecipitation-Seq analysis of mouse trophoblast stem cells [43] are provided (supplementary material, tables S8 and S9, including supplementary material Excel workbooks S2 and S3, respectively).

Applying a minimal expression threshold (RPKM >0.5 in either derived dataset) and excluding genes located on sex-linked chromosomes, *Tead4* KD triggered extensive transcriptional changes (figure 4B,C). Specifically, 667 mRNAs were significantly upregulated >twofold (155 > 10-fold), while 604 transcripts were downregulated >twofold (92 > 10-fold). A full list of differentially expressed genes (DEGs) is available in Excel workbook S1 in the supplementary material (*p* < 0.05). *Tead4* KD caused a marked reduction in *Tead4* transcript levels, with no evidence of compensatory upregulation among other TEAD-family transcription factors; indeed, both *Tead2* and *Tead3* were significantly reduced. Correspondingly, other TE marker mRNAs, such as *Cdx2* [33] and *Gata3* [31], were downregulated, although *Tfap2c* [32] showed a significant but less than <twofold decrease. By contrast, some EPI-associated markers, including early pluripotency genes *Sox2* [23] and *Fgf4* [44,45] (but not *Nanog* [8]), were upregulated. The early PrE marker *Fgfr2* was downregulated, consistent with known inner versus outer cell expression patterns established at the 16-cell stage [44,45]. Unexpectedly, other PrE genes, such as *Gata6* [8], *Sox17* [46] and *Gata4* [47,48], were induced. Genes involved in the Hippo signalling pathway [21] (including *Amot*, *Amotl2* and *Lats1*) were significantly downregulated, consistent with feedback regulation of AMOT protein expression (figure 3G,H; supplementary material, figure S7A). Paradoxically, *Yap1* transcripts were significantly upregulated, despite reduced nuclear YAP1 protein levels in marked *Tead4* KD outer cells (figure 3B,C; supplementary material, figure S6B,C), indicating potential post-transcriptional regulation. Similarly, transcripts for another TEAD4 co-factor, *Wwtr1* [49] (*Taz*), were upregulated, possibly reflecting compensatory responses to targeted *Tead4* mRNA loss. Intriguingly and relating to the observed *Tead4* KD-induced apical domain distortions/blebs (figures 1–3; supplementary material, figures S1, S4 and S5), we observed that amongst the most significantly reduced DEGs were the cytokeratin genes, *Krt8* and *Krt18* (highlighted in figure 4C); components of the intermediate filament cytoskeleton governing cell structure and shape [50]. Overall, these findings reveal substantial transcriptomic shifts in the outer cells of 32-cell (E3.5) stage blastocysts following *Tead4* KD. We further compared the DEGs with those from a recent RNA-Seq study of wild-type versus *Tead4* CRISPR-Cas9 KO mouse embryos at the earlier E3.25 stage [42]. Of the 310 downregulated and 360 upregulated genes reported in that study, 125 (including *Krt8*) and 105, respectively, overlapped with our dataset (supplementary material, table S8). Additionally, we cross-referenced our DEGs with known TEAD4-genome interactions in mouse trophoblast stem cells (TSCs), identified via chromatin immunoprecipitation (ChIP)-Seq [43]. We found that 110 of the 604 downregulated DEGs and 78 of the 667 upregulated DEGs were associated with TEAD4 binding, with 14 and eight, respectively, located in promoter regions (supplementary material, table S9; Excel workbooks S2 and S3). While these overlaps support the identification of direct TEAD4 target genes, potentially active from the 16-cell stage onwards, the majority of transcriptional changes observed following *Tead4* KD in E3.5 blastocyst outer cells are likely driven by secondary regulatory mechanisms.

We next analysed all DEGs showing >twofold change (both upregulated and downregulated) for enrichment in gene ontology (GO) terms, using process- and function-related classifications (figure 4C; supplementary material, tables S10 and S11). Consistent with the dynamic cell behaviours observed during live-embryo imaging (figure 2; supplementary material, figures S4 and S5), the most significantly enriched GO categories were associated with actin, small GTPase activity (including association with known actin regulator genes such as *Rnd1* and *Rnd3*—atypical, constitutively active small GTPases [51] that negatively regulate cell adhesion and actomyosin stress-fibre formation [52–55], that were transcriptionally induced after *Tead4* KD), cell adhesion and morphogenesis. After narrowing the GO analyses to either upregulated or downregulated DEGs (supplementary material, figures S8 and S9; tables S12 and S13 for downregulated and supplementary material, figures S10 and S11; tables S14 and S15 for upregulated), we found that these terms were predominantly enriched among the downregulated genes. This was particularly evident for actin-related terms (e.g. ‘actin filament binding’, ‘actin filament organization’, ‘regulation of actin filament-based process’ and ‘microfilament motor activity’) and GTPase-related labels (e.g. ‘GTPase regulator activity’, ‘GTPase binding’ and ‘small GTPase-mediated signal transduction’). Cell adhesion-related terms (e.g. ‘cell adhesion molecule binding’) were enriched in both upregulated and downregulated gene datasets. We also identified significant GO terms linked to epithelial development. For instance, ‘cell-cell junction organization’ and ‘epithelial cell development’ labels were enriched in downregulated DEGs, while ‘regulation of epithelial to mesenchymal transition’ was associated with upregulated DEGs.

These findings are consistent with our observations of apical bleb formation, apical domain abscission and apparent increased intrinsic contractility driving outer cell-to-ICM reallocation in both fixed (figures 1–3; supplementary material, figure S1) and live-imaged embryos (figure 2; supplementary material, figures S4 and S5). They also suggest that *Tead4* KD induces substantial transcriptomic changes that remodel the cytoskeleton (particularly the actin network, but also intermediate filament formation) and alter the adhesive properties of early- (E3.5) blastocysts stage outer cells. This potentially facilitates their atypical internalization and implicates *Tead4* as a key homeostatic regulator of spatial positioning under normal conditions. In turn, this could ensure that specified TE cells remain correctly positioned at the blastocyst embryo surface.

### 2.5. Clonal *Tead4*-knockdown blocks outer cell intermediate filament formation, yet co-expression of recombinant *Krt8* and *Krt18* mRNAs fails to rescue blastocyst outer cell-to-ICM allocations

Cytoskeletal intermediate filaments, predominantly comprised KRT18 (type-I) and KRT8 (type-II) [56], show marked inter-blastomere variability in expression at their late eight-cell onset [57,58] and are subsequently restricted to TE/outer blasto cyst cells [59], where their asymmetric inheritance is proposed to support TE specification by stabilizing the polarized apical domain and repressing Hippo signalling [24]. Our RNA-Seq revealed robust *Krt8*/*Krt18* suppression (figure 4C), and we confirmed the absence of endogenous KRT8-containing intermediate filaments in marked outer cells of *siTead4*-treated E3.5 embryos (figure 5A–C; supplementary material, table S17). However, although outer cell-to-ICM allocation is enhanced in marked *siTead4* KD clones at E3.5 (figure 1; supplementary material, figure S1), siRNA-targeted KD of *Krt8* alone, or together with *Krt18*, did not elicit any similar allocation phenotypes (supplementary material, figure S12; tables S18a,b); *Krt*-related gene KD efficiency was confirmed by RT-qPCR analysis (supplementary material, figures S12G,I; table S16). This result indicates that loss of cytokeratin intermediate filament cytoskeletal networks alone is unlikely to be the main driver of the observed si*Tead4* KD-mediated allocations. Therefore, to test whether experimentally inducing replacement cytokeratin expression could counteract *Tead4* KD, we co-microinjected single two-cell stage blastomeres with recombinant KRT8 and KRT18 encoding mRNAs (including HA-tagged KRT8 variants) and confirmed precocious and marked clone-specific intermediate filament formation in early eight-cell embryos (supplementary material, figure S13A–D). Repeating clonal *Tead4* KD (including siNTC controls) with co-injected recombinant *Krt8/Krt18* mRNAs showed marked *Tead4* KD clones still contributed more cells to the E3.5 stage ICM (and fewer cells to outer positions) than controls, despite recombinant intermediate filament expression (figure 5D–F; supplementary material, figure S13E,F; table S19). This demonstrates that induced recombinant intermediate filament network replacement cannot rescue outer cell-to-ICM allocations caused by *Tead4* KD. Indeed, apical domain distortions/blebs persisted in such marked si*Tead4*-containing outer clones, and recombinant KRT8-containing intermediate filament networks were unusually observed in marked inner cells of both treatment conditions (figure 5E; supplementary material, figure S13F).

**Figure 5.**
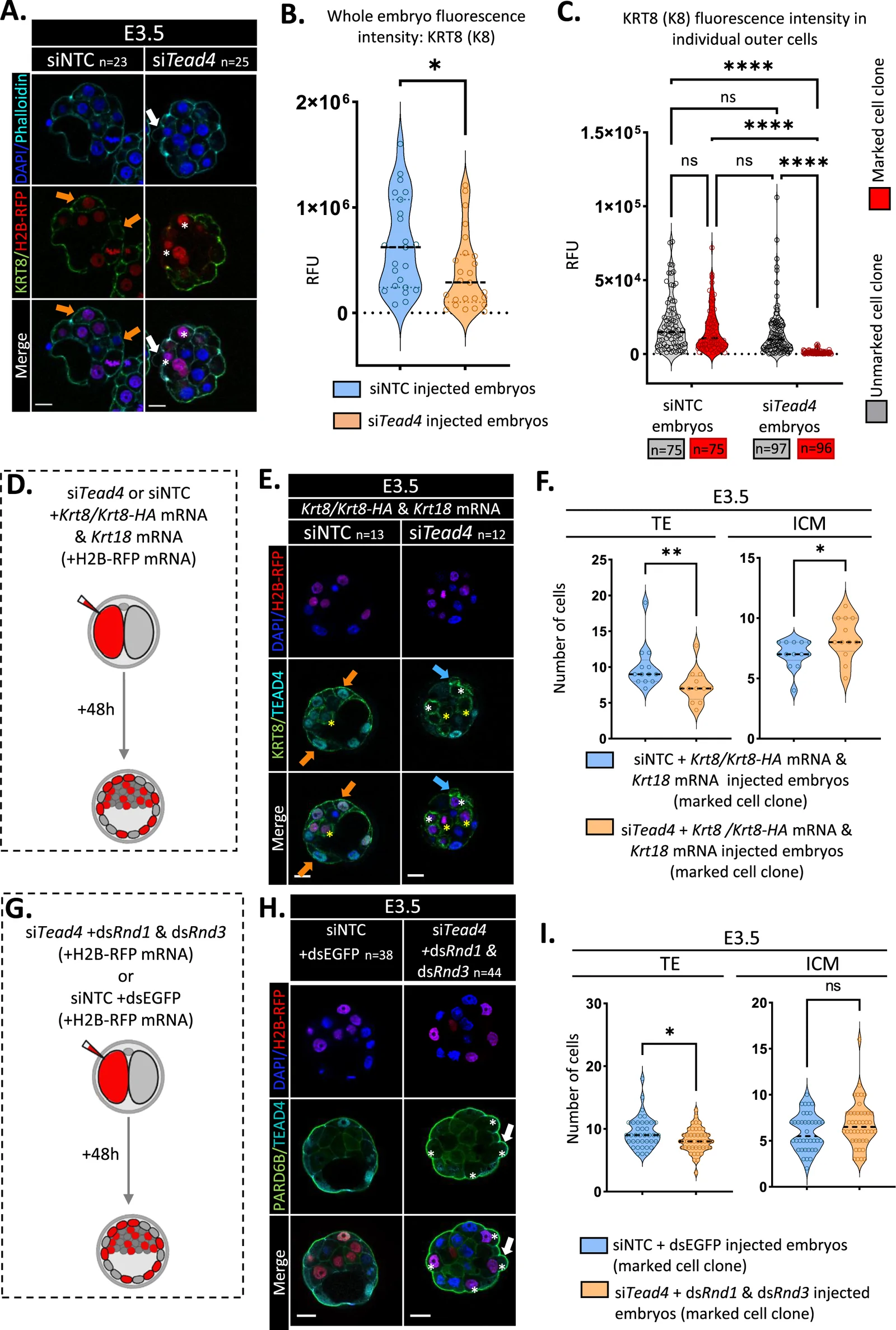
Clonal *Tead4* KD and attempted phenotypic rescue by *Krt8/Krt18* overexpression or *Rnd1/Rnd3* KD. (A) Example single z-section confocal images of E3.5 blastocysts containing H2B–RFP-marked clones (red) generated by two-cell microinjections with either siNTC (left) or siTead4 (right). Embryos are IF-stained for KRT8 (green; intermediate filaments) and counterstained with phalloidin (cyan; actin) and DAPI (blue; DNA). Scale bar, 20 µm. The number of embryos per group (*n*) is indicated. Orange arrows denote typical outer cell KRT8 expression in siNTC embryos, irrespective of clonal origin. White asterisks indicate the absence of detectable KRT8 in outer siTead4 clones, which can show abscising apical blebs (white arrows). (B) KRT8 expression across whole embryos at E3.5 (RFU, normalized), including unmarked and marked clones, in siNTC control (blue) versus si*Tead4* (orange) embryos. A significant difference (*p < 0.05) was identified between the two groups. The between-group comparison was performed using a Mann–Whitney *U*-test, selected according to data distribution. Dashed lines denote median values. (C) KRT8 expression in single outer cells (RFU) of siNTC control and si*Tead4* blastocysts; data separated for unmarked (grey) and marked (red) clones. No significant difference (*n*s, *p* > 0.05) was observed between siNTC unmarked/marked cells and the unmarked cells in si*Tead4* embryos. A significant difference (****p < 0.0001) was observed between marked cells in si*Tead4* embryos and all other cells. RFU measurements represent independent data points and are pooled according to their condition (siNTC/si*Tead4* embryos and unmarked/marked clones). Between-group comparisons were performed using Mann–Whitney *U*-tests or unpaired *t-*tests, selected according to data distribution. Median values are denoted by dashed lines. Sample sizes (*n*) are shown. (D) Schematic of the microinjection procedure to generate E3.5 early blastocysts with control (siNTC) or *Tead4* KD (si*Tead4*) marked clones, co-expressing recombinant *Krt8* (with or without an N-terminal HA tag) and *Krt18* mRNA (50% of cells) to test for phenotypic rescue of *Tead4* KD-induced clonal outer/TE-to-ICM cell allocation phenotypes. (E) As in panel A but for rescue experiments described in panel D; phalloidin actin staining is replaced by TEAD4 IF (cyan) to confirm specific KD (white asterisks). Yellow asterisks indicate enhanced recombinant KRT8 expression and intermediate filament formation in marked inner cell clones (of both treatment groups, atypical of endogenous expression). Blue arrows highlight abscised apical bleb-derived surface vesicles from outer *Tead4* KD clones (recombinant KRT8 expression data supplementary material, figure S13). (F) Outer/TE and ICM cell-number contributions of marked siNTC versus si*Tead4* clones expressing recombinant *Krt8* and *Krt18* mRNAs. Significant differences (*p < 0.05, **p < 0.01) between the two groups were identified in both lineages, suggesting that *Krt8*/*Krt18* overexpression did not rescue *Tead4* KD-induced outer/TE-to-ICM cell allocations (supplementary material, figure S13E–F). Between-group differences were assessed using Mann–Whitney *U*-tests, selected according to data distribution. Dashed lines denote median values. (G) Schematic of parallel microinjections to generate E3.5 early blastocysts with control (siNTC) or *Tead4* KD (si*Tead4*) marked clones (50% of cells), co-injecting either control dsEGFP or ds*Rnd1* iplus ds*Rnd3* constructs to attempt phenotypic rescue. (H) As in panel E but for the rescue experiments described in panel G; TEAD4 IF (cyan) replaces KRT8 staining. White asterisks denote outer-marked si*Tead4* clones, some with disrupted apical domains (white arrows). (I) Outer/TE and ICM cell-number contributions for marked siNTC/dsEGFP (blue) versus si*Tead4*/ds*Rnd1* and ds*Rnd3* (orange) clones. Significant differences (*p < 0.05) were observed in marked TE siNTC and si*Tead4* cells. No significant difference (*n*s, *p* > 0.05) was observed between marked ICM cells. Between-group differences were assessed using Mann–Whitney *U* tests, selected according to data distribution. Median values are denoted by dashed lines. Previously significant *Tead4* KD-induced increases in ICM contribution are rescued by concomitant *Rnd1*/*Rnd3* KD, but apical-domain distortions persist, indicating partial phenotypic rescue (supplementary material, figure S14A). Quantified KRT8 expression values (RFU) for panels B and C are summarized in the supplementary material, tables S17; raw clonal-allocation data are in the supplementary material, tables S19 (Krt8 and Krt18 overexpression; as in panel F) and S20 (Rnd1 and Rnd3 KD; as in panel I). Full details of the test selection are provided in §4.8. Exact *p-*values and the specific test applied for each individual comparison are provided in Excel workbook in the supplementary material.

Collectively, the data indicate TE-specific cytokeratin intermediate filaments arise as a consequence of the TEAD4-regulome, potentially via direct transcriptional regulation, but their establishment or maintenance is not essential for correct apical domain morphology or preventing outer cell-to-ICM misallocation in the blastocyst, at least under the context of clonal *Tead4* KD. Consequently, TEAD4-mediated control of TE spatial allocation likely operates through additional mechanisms, beyond KRT8 and KRT18 and intermediate filament formation.

### 2.6. Dual *Rnd1* and *Rnd3* knockdown partially rescues clonal *Tead4* knockdown-induced blastocyst outer cell-to-ICM allocation

Our RNA-Seq analysis showed clonal *Tead4* KD in outer E3.5 blastocyst cells drives robust gene expression changes enriched for GTPase- and actin cytoskeleton-related ontologies (figure 4C; supplementary material, figures S8–S11; including supplementary material, tables S10–S15). Among these were the *Rnd1* and *Rnd3* genes. As atypical small GTPases that bind GTP but lack intrinsic GTPase activity [51], *Rnd1* and *Rnd3* can act as negative regulators of actomyosin stress-fibre formation and cell adhesion by inhibiting RHOA and downstream signalling (e.g. ROCK1/2) [52–55], and in the case of human, RND3 is associated with expanding plasma-membrane blebs [52], reminiscent of marked outer si*Tead4* blastocyst clones (figures 1–3 and 5; supplementary material, figures S1 and S13). We hypothesized that the outer cell-to-ICM reallocations observed after clonal *Tead4* KD (figures 1 and 2; supplementary material, figure S1) would be sensitive to *Rnd1*/*Rnd3* expression levels. Therefore, as both the functionally related *Rnd1* and *Rnd3* transcripts were robustly induced in the outer si*Tead4* clone-derived RNA-Seq data (figure 4C; supplementary material, Excel workbook S1), and were also elevated after global *Tead4* KD by the E3.5 stage (RT-qPCR data; supplementary material, figure S14B–D; table S16—note, these data are derived from global rather than clonal *Tead4* KD, as derived in the RNA-Seq data), we targeted both for dsRNA microinjection-mediated KD (note, co-microinjected ds*Rnd1*/ds*Rnd3* global KD efficacy, versus dsEGFP negative control, was first validated at E3.5 by RT-qPCR; supplementary material, figure S14E–G).

We found that in the context of global si*Tead4*-mediated KD, ds*Rnd1*/ds*Rnd3* co-microinjection reduced the enhanced expression of *Rnd1* and *Rnd3* transcripts to levels significantly lower or equivalent to those observed in control siNTC/dsEGFP-treated E3.5 blastocysts (supplementary material, figure S14H–J; table S16). Hence, we assayed the effect of this reduction on the relative spatial allocation of si*Tead4*-marked clones (again, versus the equivalent negative technical baseline allocation of siNTC/dsEGFP microinjection controls, in which injected blastomeres received equimolar concentrations of non-specific RNAi reagents; figure 5G–I; supplementary material, table S20). We found E3.5 blastocysts containing marked si*Tead4* clones co-injected with ds*Rnd1*/ds*Rnd3* exhibited a rescue of ICM cell contribution to the technical baseline levels of control/unspecific siNTC/dsEGFP clones, reversing the enhanced ICM allocation associated with *Tead4* KD alone (figure 1; supplementary material, figure S1). However, the marked clone TE contribution was significantly reduced, suggesting only a partial rescue, as supported by the presence of apical distortions/blebs in some marked outer clones (figure 5H). The direct unmarked versus si*Tead4*/ds*Rnd1*/ds*Rnd3* marked clone comparisons, within the same embryos, showed the same trend (supplementary material, figure S14A; i.e. were comparable to the equivalent and unbiased ICM contribution in siNTC/dsEGFP-treated controls).

Overall, these data indicate that clonal *Tead4* KD disrupts the expression of the atypical *Rnd1* and *Rnd3* small GTPases, which have established links to actomyosin stress-fibre formation and plasma membrane deformation [51–55]. Moreover, this contributes to abnormal blastocyst outer cell-to-ICM allocations. They also imply that *Tead4*-driven regulation of actomyosin dynamics plays a role in the relevant spatial positioning of specifying TE cells (consistent with the cell behaviours observed in four-dimensional live-embryo imaging; figure 2; supplementary material, figures S4 and S5). Consistently, we discovered that treating late morulae/early blastocysts (E3.25) for 12 hours (i.e. spanning the period during which clonal *Tead4* KD is associated with atypical outer cell-to-ICM cell allocations; figure 1; supplementary material, figure S1) with Jasplakinolide, a potent inducer and stabilizer of actin filaments [60], resulted in the formation of outer cell polarized apical-domain distortion/blebs (containing PARD6B) and a failure to accumulate nuclear YAP1, with reduced levels equal to those observed in outer-residing marked *Tead4* KD clones at the equivalent developmental stage (supplementary material, figure S15; table S21c,d). These phenotypes are highly reminiscent of those elicited by clonal *Tead4* KD and support the existence of a broad link between actomyosin organization, apical-domain homeostasis and the correct spatial positioning of TE-specified cells under the regulation of *Tead4*; although the precise role of *Rnd1* and *Rnd3* in regulating apical domain morphology, within the spectrum of other *Tead4*-regulated genes, as opposed to directly contributing to regulating spatial positioning (figure 5G–I; supplementary material, figure S14), is less clear.

### 2.7 Conclusions

We show that *Tead4* plays a critical, cell-autonomous role in maintaining the correct outer spatial positioning of TE-specified cells in the developing mouse blastocyst, extending beyond its overlapping functions with *Tfap2c* in establishing apical-basolateral polarity [16] and its involvement in regulated Hippo signalling and TE cell fate specification [21,22]. Clonal KD of *Tead4* disrupts apical-domain morphology, causing apical distortions that are associated with abnormal cell divisions and apical bleb abscissions; these defects, together with classical asymmetric cell division and apparent apical domain constriction, result in the ectopic formation of blastocyst ICM cells. Although apical polarity is reduced, overall apical-basolateral polarity is still intact, and lateral AMOT mislocalization is not observed, suggesting that classical Hippo signalling activation [21,22] is not the principal driver of such outer cell-to-ICM transitions, despite reductions in AMOT and nuclear YAP1 levels. RNA sequencing reveals that *Tead4* broadly regulates cytoskeletal processes, particularly actomyosin-related factors, such as *Rnd1* and *Rnd3* and cell adhesion, indicating that *Tead4* ensures proper TE cell positioning by maintaining polarized apical-domain homeostasis and appropriate actomyosin dynamics.

## 3. Discussion

The first cell fate decision in mouse preimplantation development segregates TE progenitors from the ICM by the early blastocyst stage (E3.5), with only rare later internalization events reported [7]. Our four-dimensional imaging confirms that such late events are infrequent in control embryos and occur exclusively via canonical mechanisms (asymmetric division and apical constriction). By contrast, clonal *Tead4* KD markedly increases outer-to-inner cell internalization, particularly during blastocyst maturation after the 32-cell stage. These events reflect not only a generally increased trend in canonical mechanisms, but also the significant emergence of atypical behaviours associated with apical blebbing, manifest in apical domain abscission and aberrant cell divisions (whereby the comparatively rare type II events likely represent extreme variants of type I divisions). We also attempted to examine whether internalizing *Tead4* KD clones originated preferentially from mural TE (mTE) or polar TE (pTE) regions. However, interpretation was complicated by reduced cavity expansion in si*Tead4*-treated blastocysts and a tendency for cavities to form around unmarked clones, consistent with cavity-failure phenotypes reported in zygotic *Tead4* mutant mice [25]. Despite these limitations, we found no clear evidence of internalization from the mTE. Instead, internalizing clones appeared to arise from the pTE or the pTE–mTE boundary. However, this conclusion remains tentative owing to these experimental constraints. Together, these data identify *Tead4* as a key regulator of TE positioning/maintenance and apical domain stability.

Consistent with established models of TE-specification, increased clonal internalization around E3.5 in *Tead4* KD embryos correlates with Hippo-mediated fate transitions, evidenced by increased *Sox2* and reduced *Cdx2/Tfap2c* gene expression [21–23]. However, the persistence of internalization beyond E3.5, when TE fate is normally fixed [61], indicates a disruption of mechanisms that maintain outer cell positioning. Supporting this, marked cells with comparatively low CDX2 levels preferentially contribute to the ICM in chimaeras [61]. Notably, experimental KD of other TEAD4-related TE regulators (e.g. *Tfap2c* [32] or *Cdx2* [25,26] itself) does not phenocopy this effect, indicating a specific role for *Tead4* in cell spatial regulation during blastocyst maturation.

Importantly, *Tead4* KD does not abolish polarity establishment but does compromise its maintenance. Outer cells retain apical PARD6B and AMOT proteins, albeit at reduced levels. This may reflect indirect regulation via TFAP2C [32], but the observed reductions in polarity components, nuclear YAP1 and transcript levels of Hippo-related genes support a *Tead4*-dependent feedback mechanism reinforcing both polarity and TE identity. Such reduced polarity may lower the threshold required to trigger internalization, for example, by increasing susceptibility to asymmetric division [7] or actomyosin-driven apical constriction [11,12, 18–20,35–38] (as described within the ‘polarity-dependent spatial positioning’ model [28]). However, the absence of ectopic lateral AMOT localization argues against classical polarity-loss-induced Hippo activation [21,62], suggesting instead that *Tead4* maintains spatial homeostasis through combined effects on polarity and its broader transcriptional programme.

A key finding is the emergence, during blastocyst maturation, of increased internalization of marked *Tead4* KD clones, particularly via undescribed atypical mechanisms, including apical bleb-associated divisions and division-independent apical abscission. The presence of PARD6B within apical blebs and their vesicular remnants indicates that these processes involve resection of polarized, albeit compromised, apical domains [29,30]. This is consistent with impaired cortical integrity required for stable surface positioning, likely reflecting *Tead4*-dependent transcriptional regulation. Indeed, transcriptomic analysis further implicates widespread dysregulation of cytoskeletal genes, particularly those linked to actin dynamics. Although cytokeratin genes (*Krt8/Krt18*) are robustly downregulated by *Tead4* KD, direct functional assays indicate this is not a primary driver of the phenotype (despite their reported asymmetric inheritance by outer TE progenitors and roles in stabilizing apical polarity and *Cdx2* expression [24]). Instead, actomyosin regulation appears central, supported by the enriched GOs described and the recapitulation of key *Tead4* KD phenotypes (including PARD6B-positive apical blebbing and reduced nuclear YAP1) upon Jasplakinolide treatment in early blastocysts. This aligns with developmentally earlier roles for *Tead4* (with *Tfap2c*) in establishing polarity via actin remodelling at the eight-cell stage [16,17], but here indicates a later, *Tfap2c*-independent function of *Tead4* in maintaining spatial organization in the blastocyst. Consistent with this, elevated expression of *Rnd1* and *Rnd3*, GTPases that regulate actomyosin dynamics and membrane blebbing [51–55], contributes to the phenotype, as co-depletion with *Tead4 KD* rescues excessive internalization, but without affecting apical blebbing. Such incomplete rescue suggests that *Tead4* regulates blastocyst TE cell positioning through multiple pathways, including cytoskeletal, cell adhesion and morphogenetic programmes (as implied in our ontological analysis). Collectively, these findings align with general observations that increased actomyosin contractility drives internalization of apolar cells during the developmentally earlier cleavage stages [11,12,18,55]. Our live imaging of *Tead4* KD clones also supports this, revealing similar apical constrictions, including apical blebbing and abscission events preceding cell internalization, consistent with enhanced actomyosin contractility. Together, this supports a model in which actin dynamics remain a key component of cell positioning throughout blastocyst maturation itself.

Interestingly, our four-dimensional time-lapse imaging and lineage-tracing analyses did not uncover significant differences in the average number of cell divisions, either overall or within TE/outer and ICM populations, between marked *Tead4* KD and siNTC control clones/embryos (supplementary material, figure S16A,B; table S22a,b), indicating that proliferative effects are not a component of reported phenotypes. However, it is possible that PARD6B-positive apical blebbing may indicate induction of stress- or apoptosis-associated processes linked to epithelial cell extrusion [63,64]. Consistent with this interpretation, we found a significant increase in apoptotic events in marked *Tead4* KD clones compared with siNTC controls (*p < 0.05; supplementary material, figure S16C; table S22c). These findings are consistent with the reduced overall contribution of *Tead4* KD cells to late- (E4.5) blastocyst cell numbers, despite their relatively increased contribution to the ICM as blastocyst development progresses. Although apoptosis showed an increasing trend in marked outer *siTead4* clones and in ICM clones that were already internalized at the start of imaging (having arisen during preceding, non-imaged cleavage stages), neither differed significantly from their siNTC counterparts. By contrast, apoptosis was significantly elevated in ICM cells within imaged internalizing *siTead4* KD clones, but importantly not events associated with atypical apical blebbing-mediated internalization (which were negligible). Instead, apoptosis significantly followed conventional internalization via asymmetric division, with a similar but non-significant trend observed following cell constriction events (supplementary material, figure S16D; table S22d). However, the timing of apoptosis did not differ significantly between siNTC- and *siTead4*-treated embryos, regardless of cell origin (supplementary material, figure S16E; table S22e,f), with most events occurring around the mid-blastocyst stage, consistent with reports of normal, unmanipulated development [7]. Therefore, although *Tead4* KD promotes atypical, apical blebbing-associated internalization, these cells are not preferentially eliminated, contrary to observations in other epithelial contexts [63,64]. Rather, apoptosis predominantly affects cells internalized via conventional mechanisms during blastocyst maturation. This may reflect an exacerbation of reported selective ICM cell competition and apoptotic elimination that accompanies blastocyst maturation and has been associated with reduced ‘TEAD activity’ and lower nuclear YAP1 levels (primarily attributed to *Tead1*, but potentially involving *Tead2*, *Tead3* and the targeted *Tead4*) [65]. Consistent with this, our RNA-Seq analysis showed significant downregulation of *Tead2* and *Tead3*, but not *Tead1*, in marked outer/internalizing *Tead4* KD clones at the E3.5 blastocyst stage. Notably, despite increased apoptosis, the ICM remained enlarged up to E4.5 owing to the increased contribution of *Tead4* KD cells. Such cells are also reported to exhibit a bias toward EPI over PrE fate [27], indicating retention of pluripotent characteristics. This suggests that surviving *Tead4* KD ICM cells preferentially arise through later, non-canonical internalization mechanisms during blastocyst maturation, including apical abscission (figure 2E).

The presence of apical blebs may also reflect a mechanical imbalance between actomyosin-driven forces and opposing polarity maintenance (albeit compromised) [11,12,18,28]. As such, cell internalization may only occur when contractile forces exceed a counteracting polarity-dependent threshold. In this model, *Tead4* would primarily constrain actomyosin behaviour, with supportive effects on polarity, to maintain the spatial integrity of the TE. Loss of *Tead4* expression would disrupt this balance, allowing cortical instability to drive both canonical and noncanonical cell internalization events, representing a functional uncoupling of polarity from positional stability that deviates from the polarity-dependent positioning model [28] of unperturbed development.

In summary, *Tead4* not only initiates apical-basolateral polarity and TE specification but is also key to maintaining TE spatial organization during blastocyst maturation. This function is mediated primarily through transcriptional regulation of cytoskeletal dynamics, particularly actin-related pathways, alongside feedback on polarity and Hippo signalling (summarized in figure 6). Identifying the key downstream effectors of this *Tead4* regulome will be essential to understanding how robust lineage segregation and spatial homeostasis are maintained in the developing mouse blastocyst embryo.

**Figure 6.**
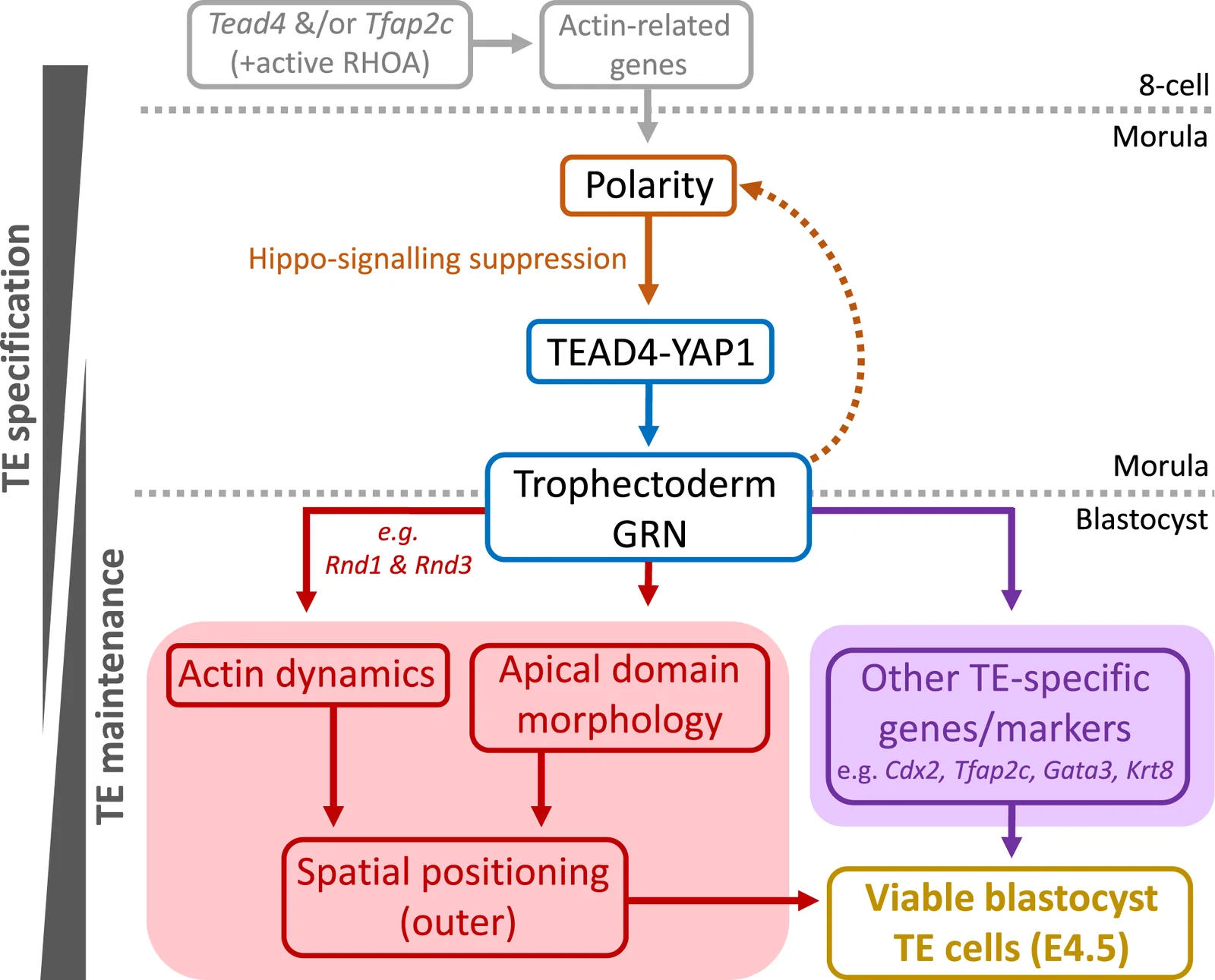
A proposed model of *Tead4*-mediated apical-domain homeostasis and regulation of relative cellular positioning within the emerging mouse blastocyst. At the eight-cell stage, *Tead4* (in partial redundancy with *Tfap2c*) activates genes involved in actin remodelling and, with active RHOA signaling, initiates apical-basolateral polarity [16]. Inherited polarity in outer cells at the morula stage contributes to suppression of the Hippo signalling pathway, leading to the formation of nuclear TEAD4–YAP1 transcriptional complexes [21,22] that initiate the TE gene regulatory network during TE specification; e.g. activating the expression of recognized TE-specific marker genes by the early- (E3.5) blastocyst stage (highlighted in purple). Simultaneously, TEAD4–YAP1 complexes promote appropriate expression of genes that maintain outer cell apical domain morphology and actin dynamics (e.g. *Rnd1* and *Rnd3*), collectively ensuring that TE-specified cells remain on the surface during blastocyst maturation (highlighted in red), with limited feedback to apical-basolateral polarity *per se*. Thus, *Tead4* plays a sequential role in TE specification and then TE maintenance during the transition from the morula to the eventual peri-implantation (E4.5) blastocyst stage.

## 4. Material and methods

### 4.1. Superovulation, embryo isolation and culture (including Jasplakinolide treatment)

All animal work complied with Czech Act No. 246/1992 under the Central Commission for Animal Welfare (approval ID 51/2015). Preimplantation-stage embryos were derived as previously described [27]. Eight-week-old F1 hybrid females (C57BL/6 × CBA/W) intraperitoneally received 7.5 IU PMSG, followed 48 hours later by 7.5 IU of hCG, and were mated overnight with F1 stud males. Embryos expressing membrane-associated fluorescent Tomato reporter were derived in similar crosses using heterozygous F1 mT/mG stud males (Jackson Laboratory, stock 00757631 [34]—with fluorescence microscopy confirmed mT expression in tail biopsies), although 5 IU of PMSG and hCG were used in the superovulations. From oviducts, superovulated two-cell stage embryos (E1.5; 46 hours post-hCG) were micro-dissected and washed through five 20 µl drops of prewarmed M2 medium (containing 4 mg ml^–1^ BSA). Unless proceeding to immediate microinjection (see below), embryos were transferred through fifteen KSOM+AA media drops (EmbryoMax; Millipore) and cultured (at 37°C in 5% CO_2_ in the terminal drop) to the desired developmental stage; note, all 35 mm culture plates were pre-equilibrated for 4 hours and were overlaid with 1.5 ml of mineral oil (Irvine Scientific). Some embryos cultured to the E3.25 stage (i.e. 42 hours post-two-cell stage recovery) were exposed to similar culture in 15 nM Jasplakinolide (Abcam), or an equivalent volume of DMSO solvent vehicle control, containing KSOM+AA drops for 12 hours (i.e. from E3.25–E3.75). For light-sheet live-cell imaging, embryos were isolated in M2 medium and cultured in global (LGGG-020, Origio) supplied with 1 mg ml^–1^ BSA at 37°C in 5% CO_2_ and 5% O_2_.

### 4.2. Two-cell stage RNAi (siRNA and dsRNA) and CRISPR-Cas9 embryo microinjections

To induce gene-specific dysregulated expression, preimplantation mouse embryo microinjections were performed with in-house prepared filamented borosilicate needles (Harvard Apparatus and Narishige) as previously described [27]. Specific details of the injected constructs are in the supplementary material, table S23. Briefly, recovered superovulated two-cell embryos were co-microinjected with indicated combinations of control or gene-specific siRNA, dsRNA, sgRNA and, as appropriate, recombinant mRNAs (e.g. encoding fluorescent Histone H2B markers, Cas9 endonuclease, KRT8 and KRT18; or non-genetically encoded RDBs, as an alternative lineage marker for RNA-Seq). To generate clones comprising 50% of the embryo (e.g. cell lineage studies), a single blastomere was injected. To assess specific gene KD/KO efficacy by RT-qPCR (and assay TE-associated gene expression), both blastomeres were injected (without lineage-marker constructs). Injections were performed in M2+BSA media drops under mineral oil on a 37°C heated stage using an Olympus IX71 microscope, manual Leica micromanipulators and an Eppendorf FemtoJet injector. Regarding CRISPR-Cas9 targeting of the *Cdx2* gene, minor modifications to a published protocol [66] were employed. After successful microinjection, embryos were cultured in KSOM+AA drops to the desired stage; non-injected embryos (one to three per experiment) served as culture control sentinels. For light-sheet live-cell imaging, single blastomeres were microinjected in M2 medium with approximately 10 pl of siNTC or si*Tead4* (10 µM) and H2B-Venus mRNA (40 ng µl^‒1^) using a PM2000B microinjector (MicroData Instrument).

### 4.3. Derivation of microinjected dsRNA and recombinant mRNA constructs

dsRNA constructs targeting murine *Tfap2c*, *Rnd1* and *Rnd3*, or enhanced green fluorescent protein as a negative/non-targeting control (or mouse *Tead4* for RNA-Seq), were designed with the ERNAi design tool [67] under default settings and produced in-house from T7 promoter-linked PCR products derived from E3.5 blastocyst cDNA templates, using the MEGAscript T7 kit (ThermoFisher). dsRNA integrity was checked by agarose gel electrophoresis; primer sequences are provided in the supplementary material, table S23.

Recombinant *Krt8* and *Krt18* (including HA-epitope-tagged *Krt8-HA*) mRNAs were generated via T3 RNA polymerase-mediated IVT from in-house cloned E3.5 blastocyst-derived cDNAs, inserted into *SfiI*-linearized pRN3P [68] plasmid vectors (which incorporate frog beta-globin UTRs for stability [69]); according to provided IVT and polyadenylation protocols (ThermoFisher; mMESSAGE mMACHINE T3 and poly-A-tailing kit). PCR cloning primer sequences, used to derive cDNA inserts, are provided in the supplementary material, table S23. mRNAs encoding Histone H2B–RFP/YFP were similarly produced from published *SfiI*-linearized pRN3 templates [70]. For CRISPR-Cas9 targeting of *Cdx2*, recombinant polyadenylated *Cas9* mRNA (in this case, *Cas9* fused to monomeric streptavidin; *Cas9-mSA*) was synthesized from *NotI*-linearized pCS2+Cas9-mSA plasmid [66] (Addgene #103882) template, using the mMESSAGE mMACHINE SP6 kit (ThermoFisher). The integrity of all recombinant mRNAs was confirmed by denaturing agarose gel electrophoresis.

### 4.4. RNA-seq of marked ds*Tead4* and unmarked E3.5 stage blastocyst outer cell clones

Per biological replicate, 10 two-cell stage mouse embryos were co-microinjected in single blastomeres with *Tead4-*specific dsRNA (ds*Tead4*, prepared in-house) and RDBs and cultured to the 32-cell stage (E3.5—apical bleb formation was visually confirmed on a stereo-dissecting microscope). The *zona pellucidae* were removed in 20 µl drops of 37°C Acid Tyrode's solution (ThermoFisher), and the blastocysts were then transferred to 20 µl drops of prewarmed, BSA-free M2 media containing 1 : 100 diluted YGMs (0.20 µm YGMs; Fluoresbrite YG, Polysciences), incubated for 1 minute at 37°C/5% CO₂. They were then washed through 10 drops of BSA-free M2 media lacking YGMs, and outer cell endocytic YGM labelling was confirmed on an IX71 (Olympus) inverted fluorescence microscope. The labelled blastocysts were then transferred to prewarmed 20 µl Ca^2^⁺/Mg^2^⁺-free M2 drops and disaggregated into single blastomeres by repeated pipetting; after which, two groups of blastomeres were collected (YGM-labelled outer cells from the RDB marked/labelled *Tead4* KD clone, and outer cells only labelled with YGMs representing the unmarked control outer cell clones). The pooled cell populations were placed into 2 µl RNA isolation buffer (PicoPure RNA Isolation Kit; Arcturus Biosciences), flash-frozen in liquid N_2_ and stored at −80°C (note, the inner cell populations were not collected). Using this methodology, a total of 10 biological replicates were prepared. Per biological replicate, the number of RDB-marked outer *Tead4* KD cell clones/unmarked control outer clones was as follows: 54/89, 65/76, 85/118, 78/114, 97/77, 89/98, 63/91, 94/124, 52/75 and 47/75. Next, the marked *Tead4* KD and unmarked outer clone-derived isolation buffer lots were pooled (20 µl each), and RNA was purified and eluted in 9.5 µl HPLC-grade water, as per the manufacturer's instructions (PicoPure RNA Isolation Kit), before preparing duplicate RNA-Seq libraries, as previously described [71], using the NEBNext Ultra II RNA Library Prep Kit from Illumina and the NEBNext poly(A) mRNA Magnetic Isolation Module, according to the manufacturer's instructions. Libraries were sequenced with 50 bp single-end reads on the HiSeq4000 platform, yielding 38–57 million reads per library. Data were then trimmed using TrimGalore! v0.6.1 and mapped to the mouse GRCm38 genome using Hisat2 v2.1.0. Gene expression (RPKM) was measured in Seqmonk v1.47.2. DEGs were identified using DESeq2 (genes on X and Y chromosomes were excluded, and a minimum per-gene RPKM expression threshold of >0.5 was applied in relation to gene expression within at least one of the clone datasets). GO analysis was performed using the clusterProfiler package in R. Datasets are available in the GEO database under accession number GSE346221. The list of identified significant DEGs (>twofold) was compared with similar RNA-Seq-derived DEG lists from the report of Wu *et al.* [42], detailing transcript changes in whole-embryo *Tead4* genetic KOs, after CRISPR-Cas9 gene editing, at the E3.25 stage. For DEG comparison with TEAD4 ChIP-Seq data from mouse TSCs derived by Home *et al.* [43], raw data were trimmed using TrimGalore! v0.6.1 and mapped to mouse GRCm38 genome using Bowtie2 v2.3.5. Peaks were then called using MACS2 within Seqmonk v1.47.2. and filtered against identified DEG inputs.

### 4.5. Immunofluorescent staining and confocal microscopy

Prior to embryo fixation, *zonae pellucidae* were removed in acid Tyrode's solution (Sigma-Aldrich, T1788) followed by sequential M2 washes. Fixation and IF staining were performed in 96-well plates (Greiner, 655101) at room temperature (unless otherwise stated). Embryos were fixed in 4% paraformaldehyde (ThermoScientific; J61899.AK) for 20 minutes, followed by several brief 0.15% phosphate-buffered saline with Tween 20 (PBST) washes and a terminal 20-minute incubation (0.15% Tween 20; Sigma-Aldrich, P1379, in 1× PBS; Gibco, 70011-036). For cell membrane permeabilization, embryos were transferred to 0.5% TritonX-100 (Sigma-Aldrich, T8787) in 1× phosphate-buffered saline (PBS) for 15 minutes and washed in 0.15% PBST, as above. For non-specific epitope blocking, embryos were incubated in 3% BSA (Sigma-Aldrich, A7906, in 0.15% PBST) for 30 minutes at 4°C. Embryos were then incubated in primary antibodies diluted in BSA blocking buffer for approximately 16 hours at 4°C. Prior to secondary antibody exposure, embryos were washed in 0.15% PBST and transferred to BSA blocking buffer for 30 minutes. Embryos were incubated with fluorescently conjugated secondary antibodies diluted in BSA blocking buffer for 1 hour at room temperature before final washing in 0.15% PBST. In some cases, fluorescence-conjugated phalloidin staining was performed before mounting. Embryos were incubated in phalloidin (50× in 1× PBS) for 30 minutes at room temperature, followed by washing in 0.15% PBST for 20 minutes. Details and dilutions of the primary and secondary antibodies used are outlined in the supplementary material, tables S24. Embryos were mounted in DAPI-containing media (Vetashield; Vector Laboratories, H-1200, diluted 1:9 in 1× PBS) in glass-bottom 35 mm dishes (Mattek, P35G-1.5-14-C) on replacement cover slides (VWR International, 631-0124) overlaid with mineral oil (Sigma-Aldrich, M5904). Complete embryo fluorescent z-section series were obtained by confocal imaging using an FV10i confocal laser scanning microscope and FV10i-SW image acquisition software (Olympus), using identical acquisition settings for all control and experimental conditions in a single dataset. All images were captured at 520 × 520 image resolution. For embryonic stages up to and including E3.75, 2 µm z-stacks were obtained. For embryos at E4.0 and E4.5, 1.5 µm z-stacks were obtained.

### 4.6. Image analyses of fixed samples

Fluoview FV10-ASW 4.2 Viewer software (Olympus) was used to classify inner and outer cell populations in fixed embryo samples. Outer cell populations were distinguished from inner cell populations by the presence of a contactless apical domain, indicated by PARD6B accumulation. Protein expression levels of defined regions of interest (ROI) were quantified manually in ImageJ (Fiji [72]) using the Polygon tool or the Line tool, depending on the nature of the analyses. The relative fluorescence units (RFU) were calculated by subtracting the background calculation (from a region outside the imaged embryo) from the total fluorescence (integrated density) of each ROI: RFU = IntDen – (*A* × *X*), where IntDen is the integrated density of the ROI, *A* is the area of the ROI and *X* is the mean grey area of the background. Whole-embryo quantification was acquired by projecting the stacks using the Stk function and manually tracing around the embryo using the Polygon tool. Apical/lateral domain protein quantifications, such as for PARD6B, AMOT, CTNNB1 and KRT8, were obtained from the centre stack of a given analysed cell (or, in the case of KRT8 quantification, from the central and two z-sections above and below) using the Polygon tool to trace around the apical/lateral domains. YAP1 quantification was obtained using the Polygon tool (tracing around the given cell/nucleus) and was calculated by averaging RFU from the central three stacks of a given cell/nucleus. Cytoplasmic YAP1 RFU was quantified by subtracting nuclear YAP1 RFU from the whole cell YAP1 RFU. PCA were performed in R v. 4.4.1 [73] using RStudio [74], FactoMineR [75] and factoextra [76] packages, providing PCA functions and extraction of function data, respectively. The ggplot2 function was used to create customized graphs, the RColorBrewer tool was used for colour palette visualization of data points, and the dplyr function was used for the visualization of 3D plots. Input data were imported as CSV files; the categorical variables, outlined in the supplementary material, figures S6D and S7B, were converted into factors and further grouped into interactive terms to represent the experimental classification of each data point. The interactive terms were assigned a factor level to ensure visual representation of clusters on the final scatter plot. Prior to scatterplot generation, the eigenvalues, cumulative variance and correlation matrices were examined to determine which dimensions explain the majority of variance within the data set and which variables correlate strongly to each dimension. PCA, eigenvalue and centroid data were extracted from the data and mapped onto the final PCA scatter plot. Clusters are visualized by differential colouring of the experimental classification, including 95% confidence ellipses. The axes represent the amount of explained variance in each dimension (>70%). The full code for the AMOT/CTNNB1 and YAP1 localization/expression PCAs can be found at https://github.com/Collier0123/AMOT_CNTTB1_PCA and https://github.com/Collier0123/YAP1_PCA, respectively.

### 4.7. Live embryo time-lapse imaging and analyses

Transgenic mT^+^ [34] two-cell stage embryos were recovered into M2 media and single blastomeres microinjected with siNTC or si*Tead4* (10 µM) and H2B-Venus mRNA (40 ng µl^–1^) and cultured in global (LGGG-020, Origio) supplemented with 1 mg ml^–1^ BSA at 37°C in 5% CO_2_ and 5% O_2_ for 48 hours, as described above. Time-lapse imaging was performed using a Viventis LS1 Live inverted light-sheet microscope system (Viventis Microscopy Sarl, Switzerland), as previously described [77]. Complete embryo imaging was performed between 89 and 105.5 hours post-hCG (relative to super-ovulation). Embryos were placed in a 125 µl culture medium covered with 125 µl oil (OVOIL, Vitrolife) in a sample-holder multi-well (Viventis Microscopy). Fifty-one 3 µm optical sections were taken with a 1024 × 1024-pixel image resolution using 10-minute time intervals. Venus and mT fluorescence were excited by 515 and 561 nm laser lines, respectively. To detect Venus emissions, a band-pass filter with a 539/30 bandwidth was used. mT was detected using a triple-band-pass filter at 488/561/640. Marked clones were tracked using Imaris software (version 9.9.1, Oxford Instruments), up to 100 frames (16.5 hours). Light-sheet time-lapse imaging files are available in the BioStudies database under accession number S-BSST2271 (https://www.ebi.ac.uk/biostudies/studies/S-BSST2271). Other data are available from the corresponding author upon reasonable request. Lineage trees were generated automatically using the spots tool and edited manually. Each track was assigned a label based on its localization and/or internalization mechanism (or apoptotic events). At frame 100, inner cells originating from an internalization were recorded manually for each video (figure 2E), and each internalization event was placed into a frequency matrix (heat map) corresponding to cell stage, normalized to the sample size (*n*) (figure 2F). Full-length example time-lapse movies are provided (supplementary material, movie S1).

### 4.8. Statistical analysis

All statistical analyses were performed using GraphPad Prism (9.2.0). For comparisons between independent groups of embryos (e.g. marked siNTC versus marked si*Tead4* or unmarked siNTC versus unmarked si*Tead4* clones), unpaired tests were used. When subdividing recorded data into specific mechanisms or derived origins, we ensured statistical robustness by applying the Bonferroni adjustment to *p-*values to account for multiple tests. For cell number comparisons within individual embryos (marked versus unmarked clones), paired tests were applied. Where three groups were compared simultaneously (e.g. *Cdx2*-KO embryos with two control groups), one-way ANOVA or Kruskal–Wallis tests were applied as appropriate to avoid inflation of type I error from multiple pairwise testing.

In all cases, the choice between parametric and non-parametric tests was determined by assessment of data normality, based on Shapiro–Wilk testing. Parametric tests (paired *t-*test, unpaired *t-*test and one-way ANOVA) were applied to normally distributed data, and non-parametric equivalents (Wilcoxon signed-rank test, Mann–Whitney *U*-test and Kruskal–Wallis test) were applied to non-normally distributed data.

Gene expression comparisons from RT-qPCR experiments were treated as independent group comparisons and analysed using unpaired tests, selected according to normality.

Significance thresholds are denoted as follows: *p < 0.05, **p < 0.01, ***p < 0.001, ****p < 0.0001. Exact *p-*values and the specific test applied for each individual comparison are provided in the supplementary material Excel workbook.

### 4.9. Quantitative reverse-transcription PCR

To assess dsRNA/siRNA KD, total RNA was prepared from 30 cultured 32-cell (E3.5) blastocysts (typically microinjected in both blastomeres at the two-cell stage) using the PicoPure RNA Isolation kit (Arcturus). Eluted RNA (10 µl) was DNase I-treated (Ambion DNA-free kit) and reverse-transcribed to cDNA (30 µl) with oligo-dT priming (Superscript III, Invitrogen). Per quantitative PCR run, specific gene transcripts were measured as technical triplicates; 0.5 µl of diluted cDNA (1 : 3 in nuclease-free water) was used as template in 10 µl SYBR-Green-based reactions (mastermix - Qiagen), with gene-specific oligonucleotide primer pairs (final concentration 400 nM; sequences provided in the supplementary material, tables S25), on a Bio-Rad CFX96 cycler system (adopting default automatic threshold detection settings). Within a control/experimental sample, transcript levels were internally normalized to those for *H2afz* and fold expression changes (including s.d.) between the described groups were calculated (according to the ΔΔCt method [78]); raw data are summarized in the supplementary material, tables S2 and S16.

## Supplementary Material



## Data Availability

All references to deposited data and codes are described in §4, with relevant links and accession numbers provided. Supplementary material is available online [79].
